# Not one Brexit: How local context and social processes influence policy analysis

**DOI:** 10.1371/journal.pone.0208451

**Published:** 2018-12-17

**Authors:** Jiaqi Ge, J. Gareth Polhill, Keith B. Matthews, David G. Miller, Michael Spencer

**Affiliations:** 1 The James Hutton Institute, Aberdeen, United Kingdom; 2 Scotland's Rural College, Edinburgh, United Kingdom; University of Illinois, UNITED STATES

## Abstract

This paper develops an empirical agent-based model to assess the impacts of Brexit on Scottish cattle farms. We first identify several trends and processes among Scottish cattle farms that were ongoing before Brexit: the lack of succession, the rise of leisure farming, the trend to diversify and industrialise, and, finally, the phenomenon of the “disappearing middle”, characterised by the decline of medium-sized farms and the polarization of farm sizes. We then study the potential impact of Brexit amid the local context and those ongoing social processes. We find that the impact of Brexit is indeed subject to pre-Brexit conditions. For example, whether industrialization is present locally can significantly alter the impact of Brexit. The impact of Brexit also varies by location: we find a clear divide between constituencies in the north (highland and islands), the middle (the central belt) and the south. Finally, we argue that policy analysis of Brexit should consider the heterogeneous social context and the complex social processes under which Brexit occurs. Rather than fitting the world into simple system models and ignoring the evidence when it does not fit, we need to develop policy analysis frameworks that can incorporate real world complexities, so that we can assess the impacts of major events and policy changes in a more meaningful way.

## 1. Introduction

On 23 June 2016 the British people voted to exit the European Union (so-called ‘Brexit’). Many months before and after the vote, the media, the public and academics were engaged in heated debates and produced copious analyses to understand the possible impacts of such a major event. Most of the discussions, however, were general and at national level. Many treated Brexit as a shock to a society in equilibrium [1, 2]; whereas historic data is more likely to support the notion that society is an ever-evolving complex system with many factors and processes constantly interacting with each other [3, 4]. Different approaches to policy analysis may partially explain why they have reached very different conclusions regarding the estimated impact of Brexit so far [5].

Researchers have long been arguing for a complex system approach to policy analysis that takes into account local context and dynamics [6, 7]. Ostrom [8] and Ostrom, Burger [9] demonstrate that when dealing with complex, multi-layered, interacting and changing systems, we should never expect a universal answer deducted from simple, predictive models to be applied to all contexts; hence “no panaceas”. The evolution of institutions, rules and norms (both implicit and explicit) embedded in their local social-ecological context is essential to the dynamics of a system. Similarly, Weaver-Hightower [10] argues for a complex system approach for educational policy analysis so that it could vary by geographic location and dynamics in the local educational system. Gerrits [11] argues that the policy analysis should take into account the coevolution between policy and the system it governs in order to have explanatory power and predict unforeseeable and unintended consequences. Ge, Polhill [12] find that well-intentioned workplace travel management programs could lead to undesirable and unintended consequences when not considering local corporate culture and management structure. When the local context is not well understood, argues Ostrom [13, 14], unexpected, unintended and even disastrous outcomes may arise from national-level policy interventions.

Evidence shows that high-level national policy can have very different impacts under different local contexts. Working with organizations that promote sustainable living in the UK, Blake [15] has noticed tensions between national policies based on ‘information deficit’ theory and the local experience of a more complex relationship between individuals and local institutions, and argued for more attention to be paid to local diversity in the negotiation process. In Sweden, Jansson, Fosse [16] looked at the implementation of national public health policy in two municipalities, and found that the local perceptions and implementation of the policy varied, depending heavily on local contextual variables such as local public-health goals, the strength of political leadership and central control. Tödtling and Trippl [17] study innovation policy and show there is no “ideal policy” as the realization of such a policy would differ greatly between geographic areas.

When a major event such as Brexit happens, it can easily become the sole focus of public attention. However, as research above has shown, the impact of a national event or policy can be highly sensitive to the local context [15–17]. Therefore, rather than being the one and only driving force in an otherwise homogenous society that is in perfect equilibrium, Brexit to Britain is more like a powerful chemical added to a mixing pot where various complex and dynamic chemical reactions have already been happening all along. The impacts of Brexit will largely depend on how it will interact with these local contexts and processes.

This paper develops an empirical agent-based model to assess the impacts of Brexit on Scottish cattle farms. We identify a number of trends and processes among Scottish cattle farms that were on-going long before Brexit: the lack of succession, increases in lifestyle farming, diversification and industrialization, and the phenomenon of the “disappearing middle” characterised by the decline in medium sized farms [18, 19]. Using an agent-based model, we attempt to assess the impact of Brexit amidst the complex interactions between those pre-existing conditions and processes.

We find that the impact of Brexit is indeed sensitive to pre-Brexit conditions. In particular, we find that the process of industrialization can significantly alter the Brexit’s impact on the local beef and dairy industry. The impact of Brexit also varies largely across regions: some will be more resilient than others; some will gain from Brexit while others will lose. As for the phenomenon of the disappearing middle, we conclude that Brexit has a very marginal impact on the long-term trend, mainly because the mechanisms behind the trends such as lack of succession are more fundamental and persistent than can be reversed by Brexit. Clearly, Brexit is not the only factor here.

## 2. The disappearing middle: Trends and processes among Scottish cattle farms before Brexit

Over the past few decades, Scottish beef and dairy farms have been facing many challenges, despite the Protected Geographical Indication status of “Scotch beef”. The number of all cattle in Scotland has been in continuous decline since the 1970s: from 2.7 million in 1974 to about 1.8 million in 2016 [20]. Profitability is one of the main concerns. Despite having a premium product, Scottish cattle farms have been struggling to break even due to factors such as increasing costs, less favourable soil quality, and competition from abroad. As a result, the survival of cattle farms in Scotland relies heavily on agricultural subsidies. For example, according to the Figs reported in Farm Management Handbook in recent years [21], an average beef farm in Scotland would have made a loss without subsidy. Barnes, Schwarz [22] estimated that if the Single Farm Payment (SFP) were to be removed or reduced, as is possible in the future, a large proportion of Scottish cattle farms would become uneconomic.

### 2.1 Succession

A smooth succession from one generation to the next is the key to the survival and growth of family-run farms [23–25]. More recently this mechanism has been challenged by lack of succession in many European countries. For example, Fischer and Burton [26] conducted in-depth interviews with 22 farm families in Scotland, and concluded that the younger generation’s lack of interest in farming has been threatening the continuation of both family farming and associated rural communities. Similarly patterns can be found in multiple countries and regions throughout Europe [23]. For example, Hennessy and Rehman [27] found that in Ireland, higher educational attainment of younger generations together with increased employment opportunities outside of farming cause many younger people to leave their family farms. In Scotland, people worry that lack of succession could fundamentally alter the structure of the agricultural system and the local culture [28, 29].

The demographic change in rural areas is a global process, and literature has been studying the endogenous and exogenous forces that shape the process. Brereton, Bullock [30] summarise some emergent socio-economic and demographic trends in rural areas in countries in European Union, which include: the decline in the importance of agriculture in the rural economy; the increased influence of external factors such as capital costs and global market prices; the in-migration of retired people and people who own a second home in the countryside; foreign immigration to fulfil local labour demand; and an aging population. Across Europe and the US, research find that aging has been a prominent demographic feature in the rural areas [31–34], both due to the decline of local population, and the in-migration of retired population to rural areas.

**Fig 1:**
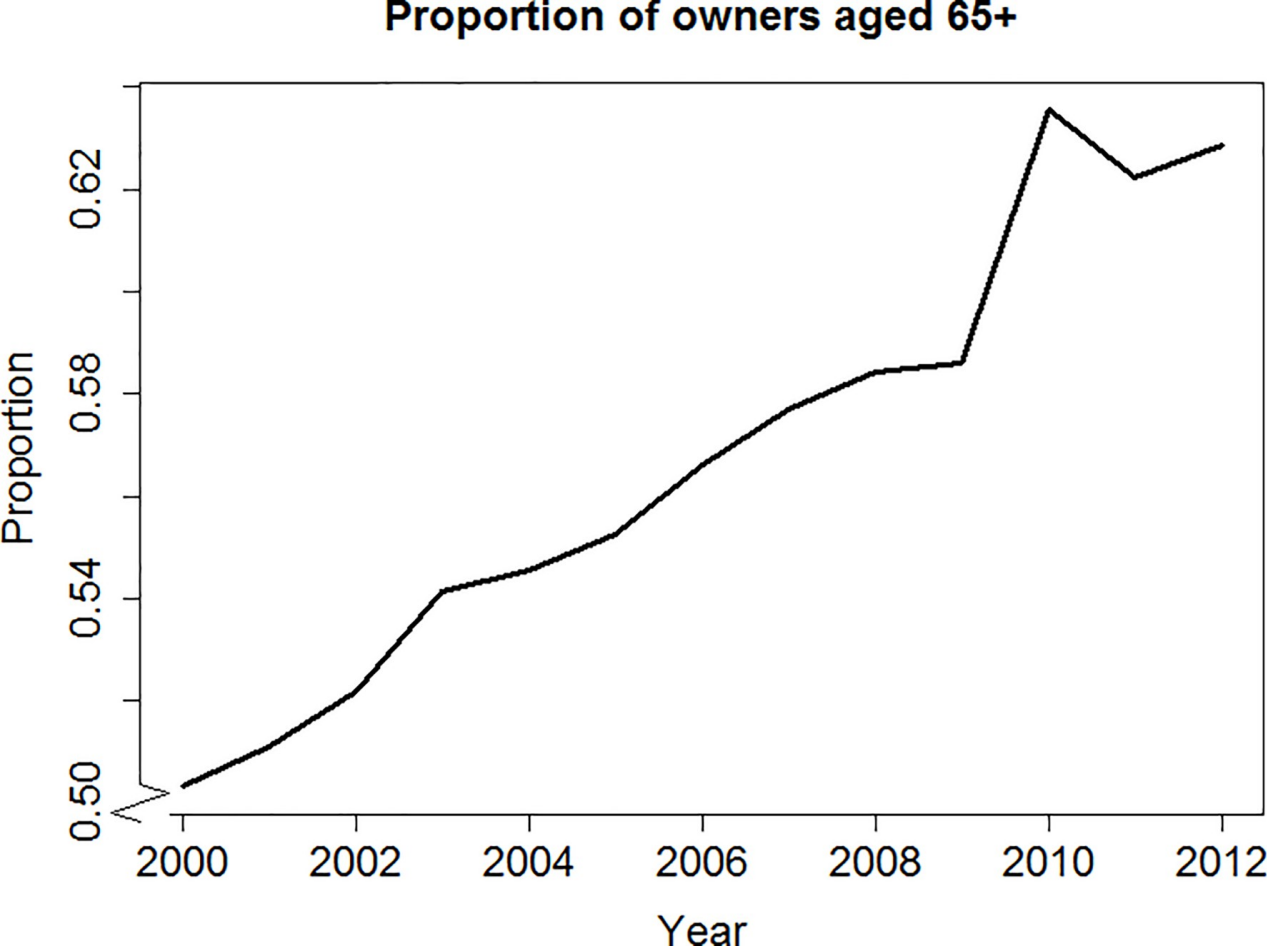
Proportion of Scottish cattle farms with owners aged 65 and more (2000–2012).

Research finds that farms who cannot identify an heir or whose owners are older and uncertain about succession tend to be smaller [35], less profitable [36], and more likely to leave farming [37]. They also show lower growth intentions and are more likely to stay stagnant [24, 25, 38, 39]. Fig 1 shows the number of Scottish cattle farms with owners aged 65 or older using the June Agricultural Census (JAC) data from 2000 to 2012. The proportion of farms whose owners are over 65 (a common retirement age) has been increasing over the years. In the absence of a successor, many will struggle when they have to retire from farming. Those who lack a successor might be forced to downsize the farm gradually and even quit farming eventually.

### 2.2 Leisure farming

Another trend in European agriculture is the rise of leisure farming, especially in peri-urban areas. An increasing number of farmers no longer see farming as their main source of income, but a way to enjoy a rural lifestyle [40, 41]. For example, Busck, Kristensen [42] found that in the Greater Copenhagen area, the importance of agriculture as the main income source in has declined significantly. The proportion of full-time farmers decreased from 26% in 1984 to 8% in 2004, with an increasing number of farmers having no prior agricultural experience. In Flanders, Belgium, Elke and Joost [43] also noticed a decrease in the number of full-time farmers and a concentration of production in a few large farms with full-time managers, accompanied by an increased demand for leisure, recreation, landscape and wildlife from the society. The rise of leisure farming has profoundly changed the structure of rural society. The conventional, production-focused view of farming and farmer behaviour will also need to change, as farmers nowadays come from a more diverse socio-economic background and often have different goals and motivations.

**Fig 2:**
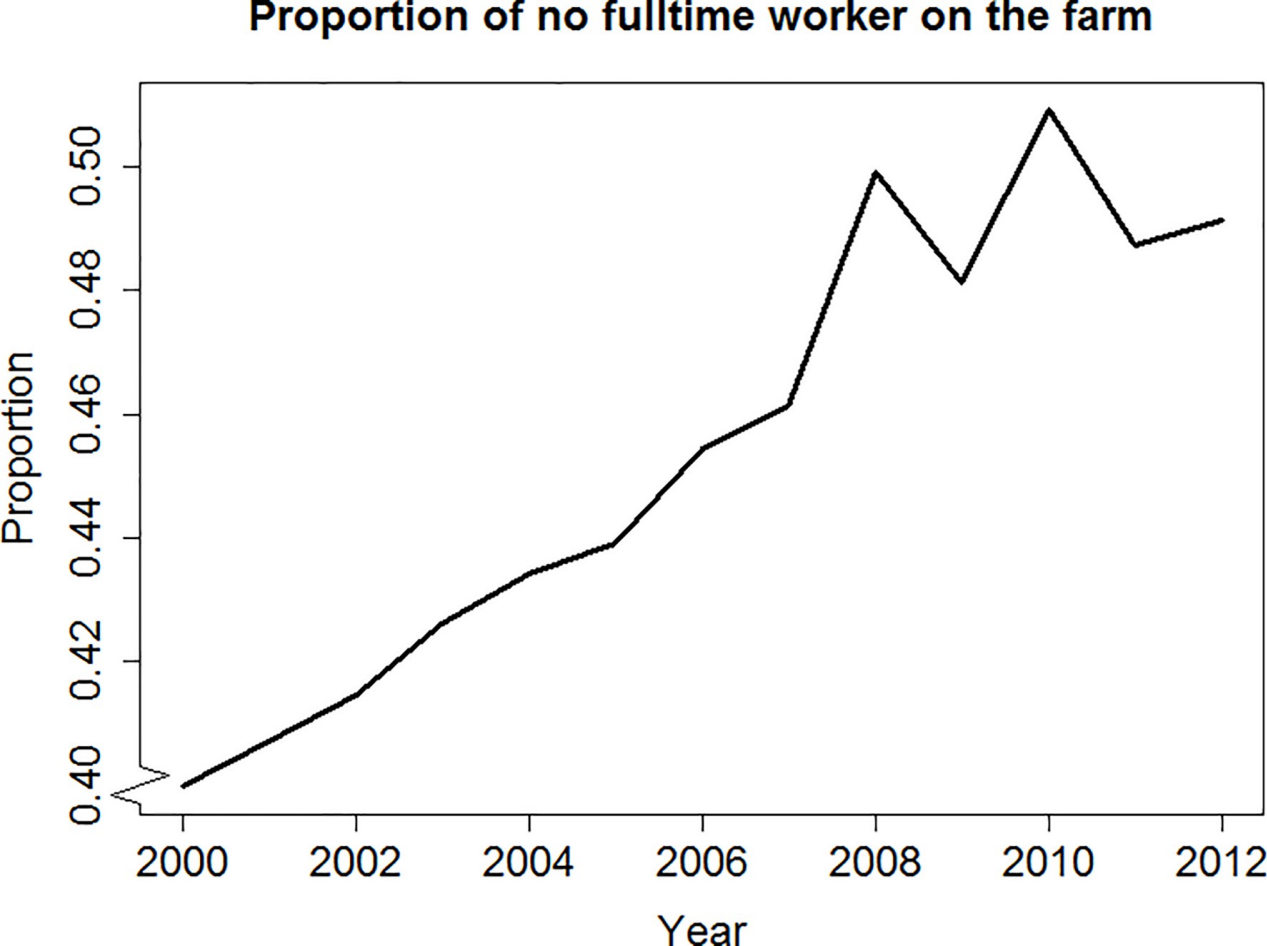
Proportion of Scottish cattle farms with no fulltime worker on the farm (2000–2012).

Zasada [44] discussed various motivations behind leisure farming, including environmental quality and landscape, leisure and recreational activities, and local food supply, particularly for high-quality and ‘natural’ products. Orsini [45] reported on a group of farmers who identify themselves as ‘hobby farmers’ in a wine producing area in Tuscany, Italy, who view farming as a way to preserve culture and lifestyle, and rely on off-farm income as their main income source. Sutherland [46] observed the return of “gentleman farmers” in the UK, who produce agricultural commodities without the intent of earning a living. The author then suggested an alternative view of farmers: as consumers of rural amenities rather than producers of agricultural products. Fig 2, drawing on June Agricultural Census data between 2000 and 2012, shows the rising proportion of Scottish cattle farms without a full-time worker on the farm (including non-agricultural activities), which we use as an indicator for the existence of off-farm income and leisure farms.

### 2.3 Diversification

At the same time, some existing farms have diversified into tourism in response to the surging demand for the leisure and recreational activities in rural areas. For some farms, agri-tourism has become an important source of income [47, 48]. Scotland has a long history of farm-based tourism, especially in the Highlands and Islands [49, 50]. Grants were offered to Scottish farmers to encourage them to diversify into tourism or other non-agricultural activities [51].

Farm tourism often displays a strong spatial clustering pattern [52]. The reason for the clustering can be manifold: farms with diversification into tourism (‘tourist farms’) tend to be located near natural attractions [53, 54] and in areas with lower soil quality [53, 55]. Being close to urban areas is found to have mixed impact on farm tourism: some found it positive [56, 57] and some found it negative [48, 55]. Moreover, farms share road infrastructure, facilities and public amenities of the area, which may lead to a positive externality, where existing tourist farms attract more new entries. Pfeifer, Jongeneel [53] find that in addition to landscape attractiveness and soil quality, a farm’s decision to go into tourism produces positive externalities: new entries tend to appear near existing ones, which partly explains the formation of tourism “hotspots” in the landscape.

To sum up, having a farm in agricultural tourism often means more facilities in the nearby area, either by it bringing in potential customers and improving the infrastructure and publicity of the area, or because farms diversified into tourism are those that locate in attractive areas in the first place, which means their neighbours are also more likely to get into tourism in the future. Either way, evidence has shown that having neighbouring farms in tourism increases the probability that a farm will diversify into tourism as well.

According to the EU Farm Structural Survey, which is conducted every 10 years on agricultural holdings in countries across the EU, 455 farms have gone into tourism between 2000 and 2010 out of 13,406 cattle farms in Scotland. Although the overall number of Scottish cattle farms in tourism increased only slightly from 682 to 709 during the same period, the data indicate that there are a lot of movements in the sector, including new entries and exits.

### 2.4 Industrialization

As globalisation integrates food supply across the world, the agricultural sector in many countries have gone through tremendous restructuring and industrialization, characterised by a manufacturing approach to agricultural production and a high degree of specialization [58, 59]. García-Arias, Vázquez-González [60] described a drastic structural change among farms in Galicia, Spain that happened in the last 20 years: from many family-run, small-scale farms to a small number of highly-specialized and geographically-concentrated farms. Research has found that farming, especially livestock farming, has exhibited efficiencies of scale, meaning larger farms can be operated at a lower unit cost. For example, Latruffe, Balcombe [61] found that in 2000, 64% of the livestock farms in Poland have increasing returns to scale. Rasmussen [62] found that between 1985 and 2006, both chicken and dairy farms in Denmark showed scale efficiency. The existence of efficiencies of scale suggests that profit-driven farms may want to expand over time to fully take advantage of them.

Farm location plays an important role in the level of scale efficiency and the tendency of farms to expand and industrialize. Dimara, Pantzios [63] found that the location of the farm significantly affects the technical and scale efficiency of conventional, non-organic farms, mainly because produce prices in some areas are consistently lower than others. Scale efficiency is also highly dependent on soil quality [64], which is spatially correlated. Distance to market is another important determinant of farm income and its potential to upscale. In addition to transport cost that is the basis of Von Thünen's model of agricultural land [65], recent research found that business links and supply chains are also highly concentrated geographically. Roberts, Majewski [66] find that agricultural transactions in Northeast Scotland are concentrated in space with both upstream and downstream agri-businesses clustered in a few places in the region. The advantages that prompt a farm to expand and industrialize, such as high-quality land and proximity to the market and agri-business centres, may well be shared by their neighbours. Moreover, the spread of agricultural technology, often associated with industrialization, also display a strong geographic concentration [67], as farmers mimic and learn from their neighbours [68].

**Fig 3:**
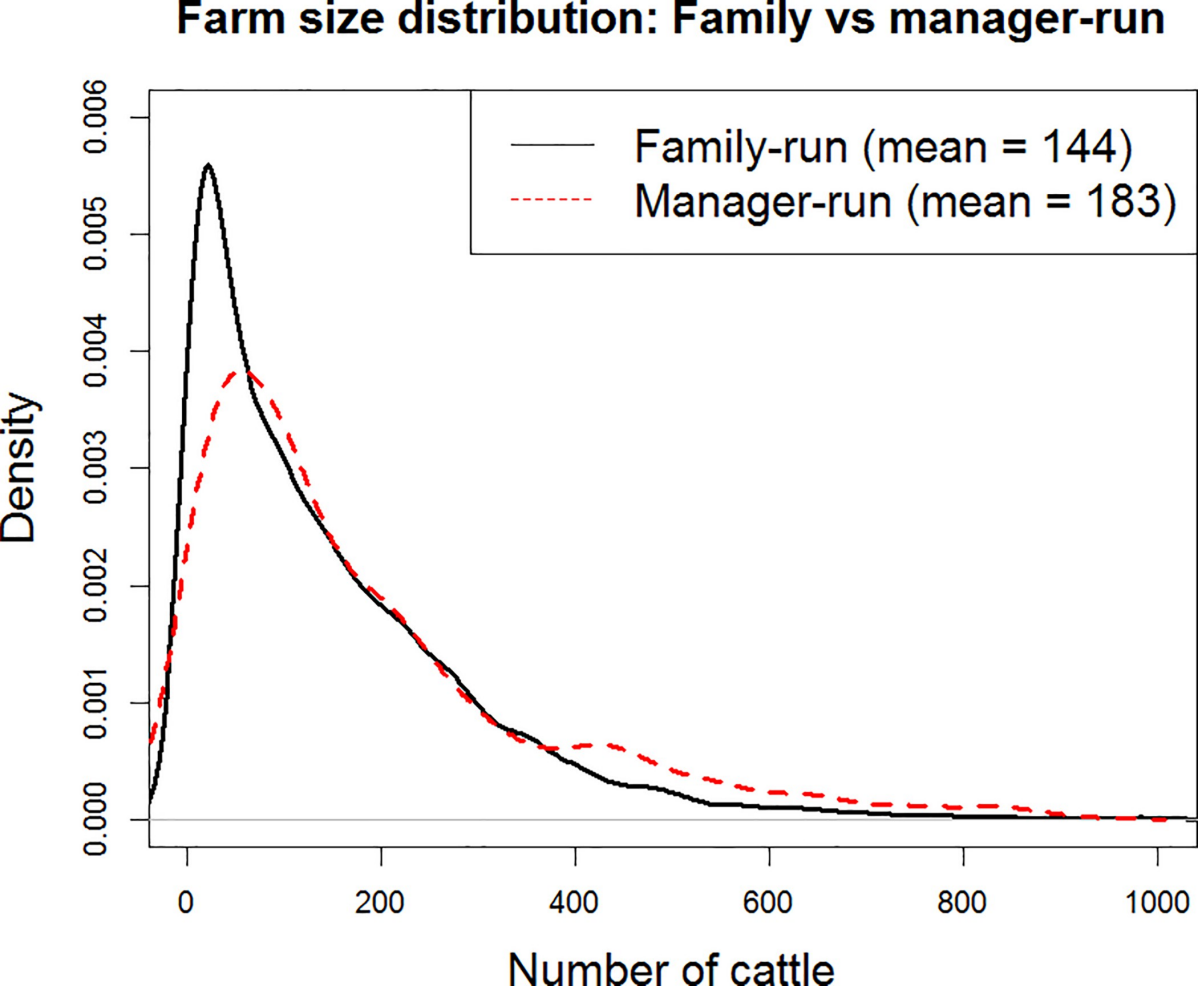
Farm size distribution (density) between family and manager-run farms; family-run farms have more farms with fewer cattle and fewer farms with more cattle than do manager-run farms.

One indicator that a farm is industrialized or has the intention and potential to do so is whether the farm is run by the owner and their family members or by a professional manager. Having a professional manager indicates that the farm has a structure that more resembles a modern business, is less constrained by the limited labour supply of family members, and is thus more capable to expand. It is also likely that when a farm reaches a certain size, a family-run structure is no longer suitable, and a professional manager will be needed to run a larger enterprise. A modern management structure may in turn enable the farm to expand further. In the June Agricultural Census data, we find that farms that are run by a manager are on average 27% larger than those run by the family. Moreover, the very large farms with more than 800 cattle are almost exclusively run by professional managers. Fig 3 shows the size distribution of family-run and manager-run cattle farms in Scotland.

### 2.5 The disappearing middle

Researchers have observed a phenomenon termed the ‘disappearing middle’ among farms, which describes an increasing polarization in farm size and the decline of medium-sized farms. The numbers of both large and small farms have been increasing, while the number of medium-sized farms has been decreasing. Slee, Brown [69] found that in Scotland, land with medium agricultural potential faces the most severe conflicts of use and competitive pressure, which the authors called ‘the squeezed middle’. Dannenberg and Kuemmerle [70] found that in post-socialist Poland, farm size distribution has polarized and medium-sized farms have declined significantly. Weiss [19] find a polarization of growth rates among Austrian farms and concluded that the current evidence supported the notion of a ‘disappearing middle’ or ‘dual distribution’. Similar patterns are found in Hungary [71] and the United States [18, 72].

**Fig 4:**
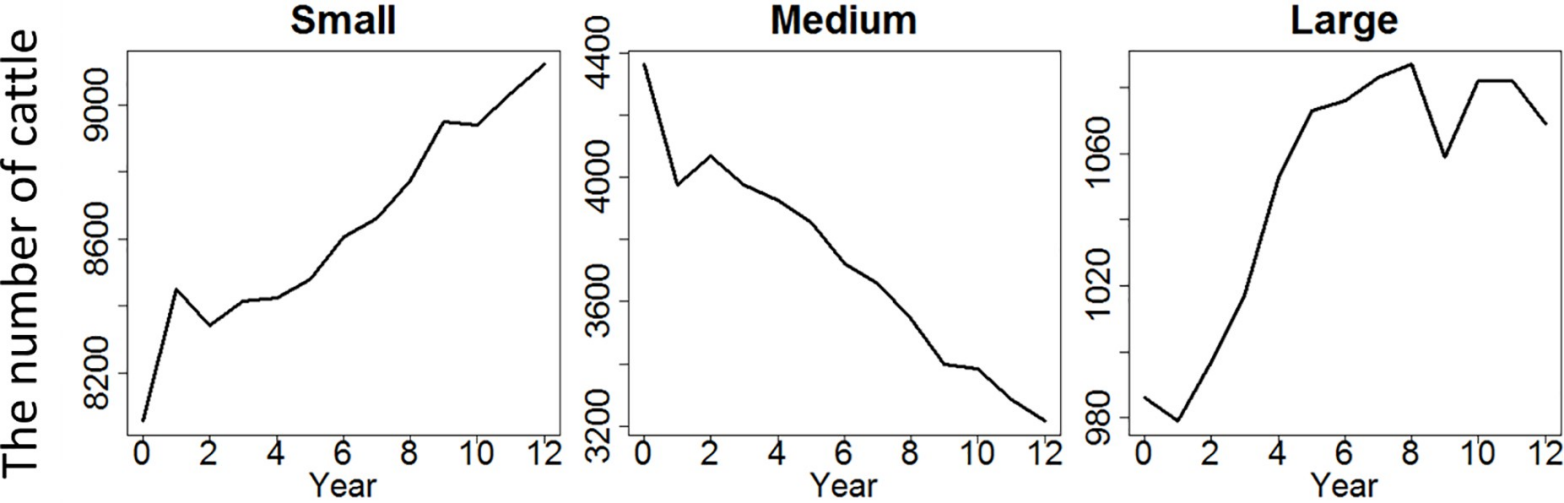
The number of small, medium and large sized cattle farms in Scotland from 2000 to 2012.

Scottish cattle farms have seen a decline of medium sized farms and a rise of small and large sized farms over the years. Fig 4 shows the number of small, medium and large cattle farms in Scotland from 2000 to 2012. To construct this Fig, we used the June Agricultural Census (JAC) data set, which is from questionnaires that are compulsory for farmers to complete and covers most agricultural holdings. There were 13,406 cattle farms in total in 2000, and the same cattle farms are used throughout the simulation. Although the number of cattle in a farm can reduce to zero during the simulation, we do not exclude them in the analysis. We do not have new entries in this model. We derived the categorization of the small, medium and large farms using a *k*-means cluster analysis (see supporting information). Between 2000 and 2012, the number of both small and large cattle farms have been increasing, while the number of medium-sized ones decreasing; hence the ‘disappearing middle’.

This study develops an empirical agent-based model of Scottish cattle farms based on individual farm and geographic data. We will explore the interactions between the above trends and social processes with various Brexit scenarios. We aim to answer the following research questions: what is the impact of various Brexit scenarios on Scottish cattle farms in midst of local context, trends and social processes? To what extent does the impact of Brexit depend on the existing conditions and mechanisms before Brexit?

## 3. An agent-based model of Scottish cattle farms

We have developed a spatial empirical agent-based model of Scottish cattle farms and farming system. The model was developed in Netlogo, a free multi-agent programmable modelling environment [73]. In this section, we will describe the model following Grimm et al.’s Overview, Design concepts and Details (ODD) protocol [74].

### 3.1. Purpose

The purpose of the model is to explore the complex dynamics behind the polarization of cattle farms in Scotland and the impact of various Brexit scenarios.

### 3.2. Entities, state variables and scales

The main entity in the model is the individual ‘agricultural holding’ (as it is termed in the JAC data), which we interpret as being a single farm. Agricultural holdings make decisions regarding herd size in each time period. There are 13,406 cattle farms identified from the June Agricultural Census database between 2000 and 2012. The census data includes information on the farm owner, herd sizes of various types of cattle, areas of crops, grasses and so on. Table 1 lists the key attributes of a Holding entity.

**Table 1:** Key attributes of a Holding entity.

Variable	Description	Exog^1^?	Const^2^?
holding code	a unique holding identifier	y	y
n beef cattle	the number of beef cattle	n	n
n dairy cattle	the number of dairy cattle	n	n
n beef calf	the number of beef calf	n	n
n dairy calf	the number of dairy calf	n	n
n beef breeder	the number of beef breeder (suckler cow)	n	n
n dairy breeder	the number of dairy breeder (suckler cow)	n	n
n service bull	the number of service bull	n	n
n other cattle	the number of other cattle	n	n
n cattle in total	the number of cattle in total	n	n
area of barley	area of barley in hectares	n	n
area of oats	area of oats in hectares	n	n
area of grass	area of grass in hectares	n	n
area of rough grazing	area of rough grazing in hectares	n	n
total area	total area in hectares	n	n
size group	small, medium, large depending on total cattle size	n	n
successor?	if the current owner has a successor	y	n
off-farm income?	if the current owner and family has off-farm income	y	n
diversified?	if the current farm has diversified into tourism	n	n
industrialized?	if the current farm has industrialized	n	n

^1^ if the attributes is exogenous

^2^ if the attributes is constant

Among other things, in each time period the owner of a holding makes decisions about the size of the herd in every category, which is encapsulated in a ‘decision entity’. Table 2 lists the key attributes of a ‘Decision’ entity.

**Table 2:** Key attributes of a Decision entity (notation same as in Table 1).

Variable	Description	Exog^1^?	Const^2^?
decision maker	the holding owner or tenant that makes the decision	n	n
decision object	the cattle type on which the decision is made. Can choose from: beef cattle, dairy cattle, beef calf, dairy calf, beef breeder, dairy breeder, service bull and other.	n	n
action	The action towards the decision object, including both the type of action (expand, shrink or status quo), and the rate to implement the action.	n	n

^1^ if the attributes is exogenous

^2^ if the attributes is constant

Spatial aggregation is done using Scottish Parliament constituency boundaries to capture the political implications of Brexit. They are the regions represented at the Scottish Parliament elections. Since the EU common agricultural policy is a devolved matter, Scottish Parliament constituencies are chosen over UK Parliament constituencies to better represent the interest of the area and its political position. There are 73 Scottish Parliament constituencies in Scotland. Table 3 lists the key attributes of a Constituency entity.

**Table 3:** Key attributes of a Constituency entity (notation same as in Table 1).

Variable	Description	Exog^1^?	Const^2^?
name	the name of the constituency	y	y
geographic area	the geographic area of the constituency	y	y
n-beef	a list, the number of beef cattle in the constituency in the previous years	n	n
n-dairy	a list, the number of dairy cattle in the constituency in the previous years	n	n
n-all-cattle	a list, the number of all cattle in the constituency in the previous years	n	n
n-holdings	the number of holdings in the constituency	y	y
n-sml	a list, the number of small cattle farms in the constituency in the previous years	n	n
n-med	a list, the number of medium cattle farms in the constituency in the previous years	n	n
n-lrg	a list, the number of large cattle farms in the constituency in the previous years	n	n
n-tourism	a list, the number of cattle farms diversified in tourism in the constituency in the previous years	n	n
n-industrialization	a list, the number of industrialized cattle farms in the constituency in the previous years	n	n
n-succession	a list, the number of cattle farms with succession in the constituency in the previous years	y	n
n-leisure	a list, the number of cattle farms with leisure income in the constituency in the previous years	y	n

^1^ if the attributes is exogenous

^2^ if the attributes is constant

### 3.3. Process overview and scheduling

Each time step in the simulation represents a year, during which the holdings will make decisions regarding their decision to diversity, to industrialize, and the decision regarding the herd size of each type of cattle (beef, dairy, cattle, calf, breeder/suckler cow etc.).

A holding’s decision to diversify into tourism is influenced by its neighbours. As we discussed in section 2.3, agricultural tourism displays strong spatial correlation and externality. In this model, we will use a probabilistic function to determine whether a farm will diversify into tourism in the next period. The probability increases as the percentage of nearby farms already in tourism increases. We define ‘nearby’ farms as those located within a radius of 10 km, which is approximately 5–10 minutes’ drive.

Industrialization is defined in this model as being run by a professional manager and expanding more quickly when conditions allow. The way we define and implement industrialization in the model is consistent with the description of industrialization in the literature, which entails the adoption of a manufacturing approach to agricultural production. A holding’s decision to industrialize depends on two factors. The first is size. We assume that a holding with fewer than 50 cattle will not consider industrialization, as it cannot sustain the wage of a professional manager. Once a farm reaches the minimum size, we assume that its decision to industrialize will depend on its neighbours. We use a radius of 15 km here because we believe information, land quality etc. can be shared on a larger spatial scale than tourism resources and facilities. Industrialized farms will expand more quickly when conditions are preferable.

Data on farm succession and leisure farms are from June Agricultural Census. We assume that farms whose owner is over 65 are likely to be without a successor. Leisure farms are defined as those with no full-time workers on the farm. We assume that for leisure or diversified farms or farms without a successor, profit from farming is not a factor for their decision-making. We assume that farms without a successor will shrink (in herd size) rapidly since its owner cannot maintain it due to old age. We assume that leisure and diversified farms will maintain the same herd size throughout the model.

**Fig 5:**
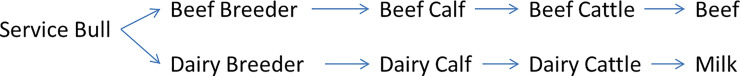
The beef and dairy supply chain.

For the profit-driven farms, we use a supply chain approach to model their decision making. The supply chain starts from service bull farmers, who supply to beef and dairy breeders, who supply to farmers who fatten beef and dairy calves, who supply to beef and dairy cattle finishers who supply the final product (beef and milk respectively) to the market. A holding may also play multiple roles in the supply chain. For example, it can own both beef calves and cattle. Fig 5 illustrates the supply chain of beef and dairy industry in Scotland.

**Fig 6:**
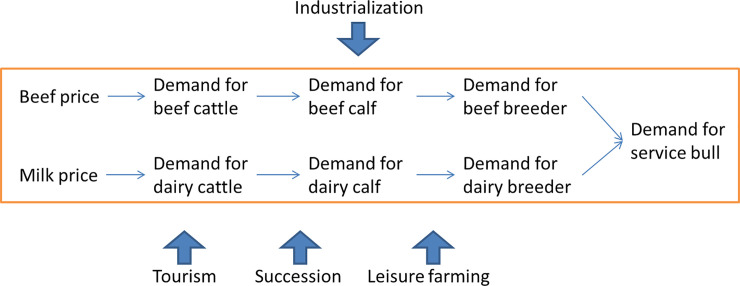
Farm decisions perpetuate from downstream to upstream in reverse order as in the supply chain (Fig 5).

Decisions are made sequentially in reverse order of position in the supply chain. The most downstream suppliers–those who sell beef and dairy cattle–are the first to make a decision on herd size based on the price of beef and milk. The decisions made by them in turn determine the aggregate demand for beef and dairy calves, which in turn determines the demand for beef and dairy breeders. The process perpetuates through the chain until it reaches the most upstream suppliers. Fig 6 illustrates the process of farm decision making perpetuating from downstream to upstream suppliers in reverse of the supply chain.

**Fig 7:**
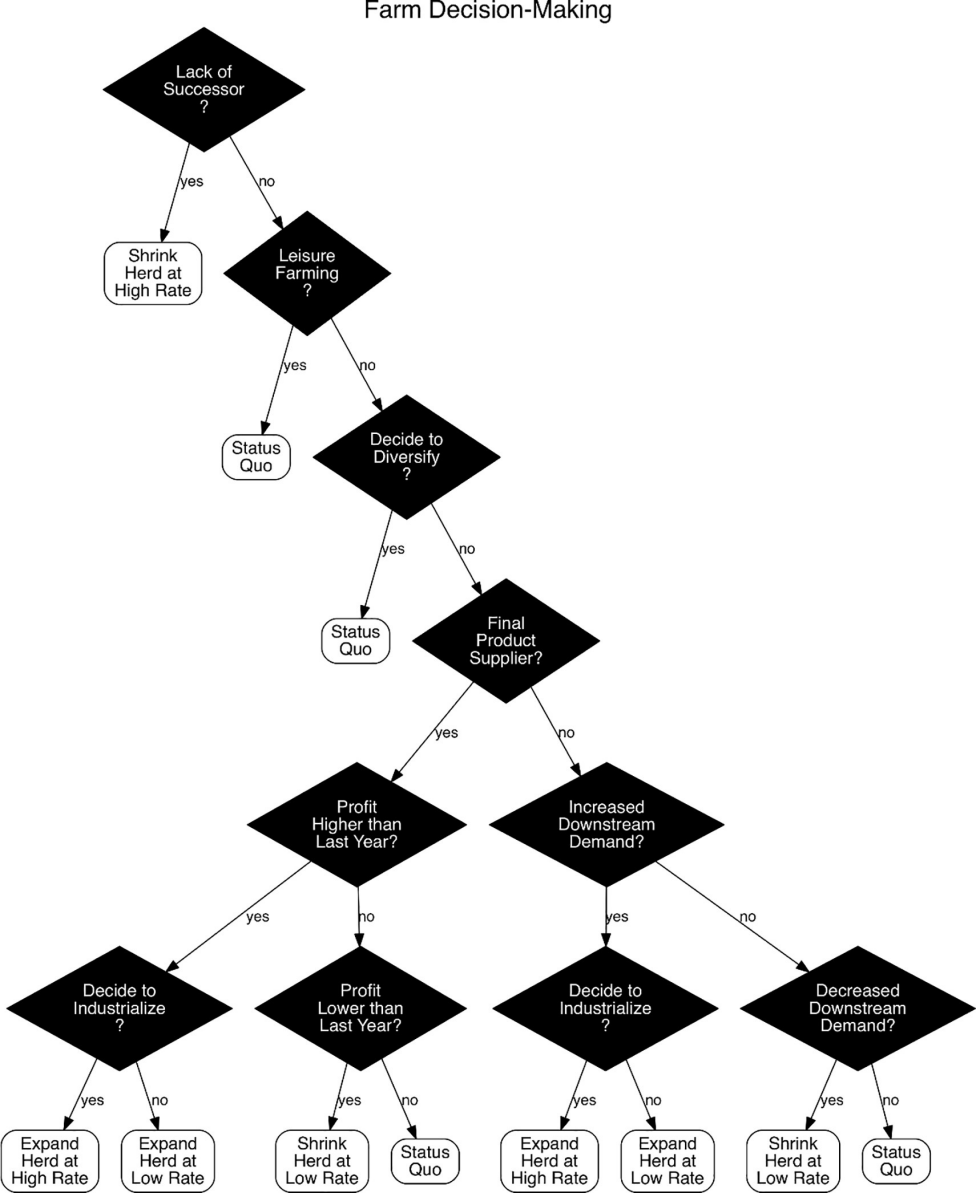
The decision making flow chart of an individual farm (status quo means remaining the same herd size, i.e., replacement = depreciation).

Fig 7 shows the decision-making flow chart of an individual farm.

### 3.4. Design concepts

#### 3.4.1. Emergence

The main emergent results are the distribution of farm sizes in Scotland, in other words, the number of small, medium and large farm respectively. The results are emergent because they are based on decisions made by individual farms. The model does not impose any constraints or rules for certain distributional patterns to occur.

#### 3.4.2. Adaptation

The individual farmers adopt different behaviour and decision rules in response to changes in themselves (e.g. whether they have succession or off-farm income), and in the environment (e.g. input and output price, the existence of tourism in the neighbourhood). The farmers are heterogenous. Based on the type and status of the farmers, only some (the profit seekers) will adapt to increase their chances of success.

#### 3.4.3. Sensing

Individual farmers sense if their neighbours have diversified into tourism or decided to industrialize or intensify their farms. They also sense the global market price for their input and output.

#### 3.4.4. Interaction

Farmers interact through observing their neighbours (in tourism and industrialization), and (with a probability) mimicking what their neighbours do.

#### 3.4.5. Stochasticity

One source of stochasticity is whether a farmer will mimic their neighbours when their neighbours diversify into tourism or decide to industrialize or intensify the farm. Another source of stochasticity is whether the owner of the farm has a successor when he or she is more than 65 years old. To account for the variability caused by the stochasticity, for each parameter combination we run the model 40 times to 1) see the level of variability and check if there are cases of bifurcation and 2) if variability from stochasticity is small to moderate and there is no bifurcation, use the average of the runs for analysis and comparison.

#### 3.4.6. Collectives

Individuals do not form collectives or aggregations in this model.

#### 3.4.7. Observation

The status and herd size of all farms are collected and then aggregated for analysis. We use all output data to imitate the empirical evidence from the census data.

### 3.5. Initialization

The data we use to initialize the model are from the June Agricultural Census (JAC) and the EU Farm Structure Survey (FSS). Both census and survey are compulsory and cover almost all farms in Scotland (see the supporting document for more information on the coverage of JAC and FSS). The data cover the time period between 2000 and 2012. The unique farm identifier also allows us to obtain the holding’s geographic location through the Integrated Administration and Control System, from which we derive each holding’s agricultural capacity from Macaulay Land Capability for Agriculture (LCA) data [75]. The integrated data provide detailed information on each holding’s land area, land quality, crops and livestock, as well as the demographics of the occupier, which we then use to construct the empirical agent-based model. Since this study focuses on cattle farms, we include only the farms with at least one head of cattle in 2000. Of the more than 40,000 farms in Scotland, we identify 13,406 cattle farms, all of which we include in this model.

The successor variable is derived from the occupier’s age in JAC. If the age of the occupier is over 65 (the current pension age for men in the UK), we assume a high proportion of them will not have a successor. At the start of the model in 2010, 1,391 (10.4%) farms have no successor. The off-farm income variable is derived from whether the occupier works full-time on the holding in JAC. If no-one (no occupier, family members or professional manager) works full-time on the holding, we assume the family has off-farm income and are leisure farmers. At the start of the model in 2010, there are 5,459 (40.7%) leisure farmers by this definition. Both successor and leisure farm status are dynamic variables from annual data and are subject to change in the simulation.

We categorize the farms into three size groups: small, medium and large by the total number of cattle. The cut-offs that we use to categorize the farms are derived from the clustering analysis using the 23 cattle-related variables in the JAC. The cut-off between small and medium-sized farm is 131 cattle, and the cut-off between medium-sized and large farm is 368 cattle. The values of the cut-off are derived from the cluster analysis on cattle farm size (see the supporting document for more information on cluster analysis) and remain unchanged throughout the simulation. At the beginning of 2000, there are 1,030 (7.7%) large holdings, 4,424 (33%) medium-sized holdings and 7,952 (59.3%) small holdings.

**Fig 8:**
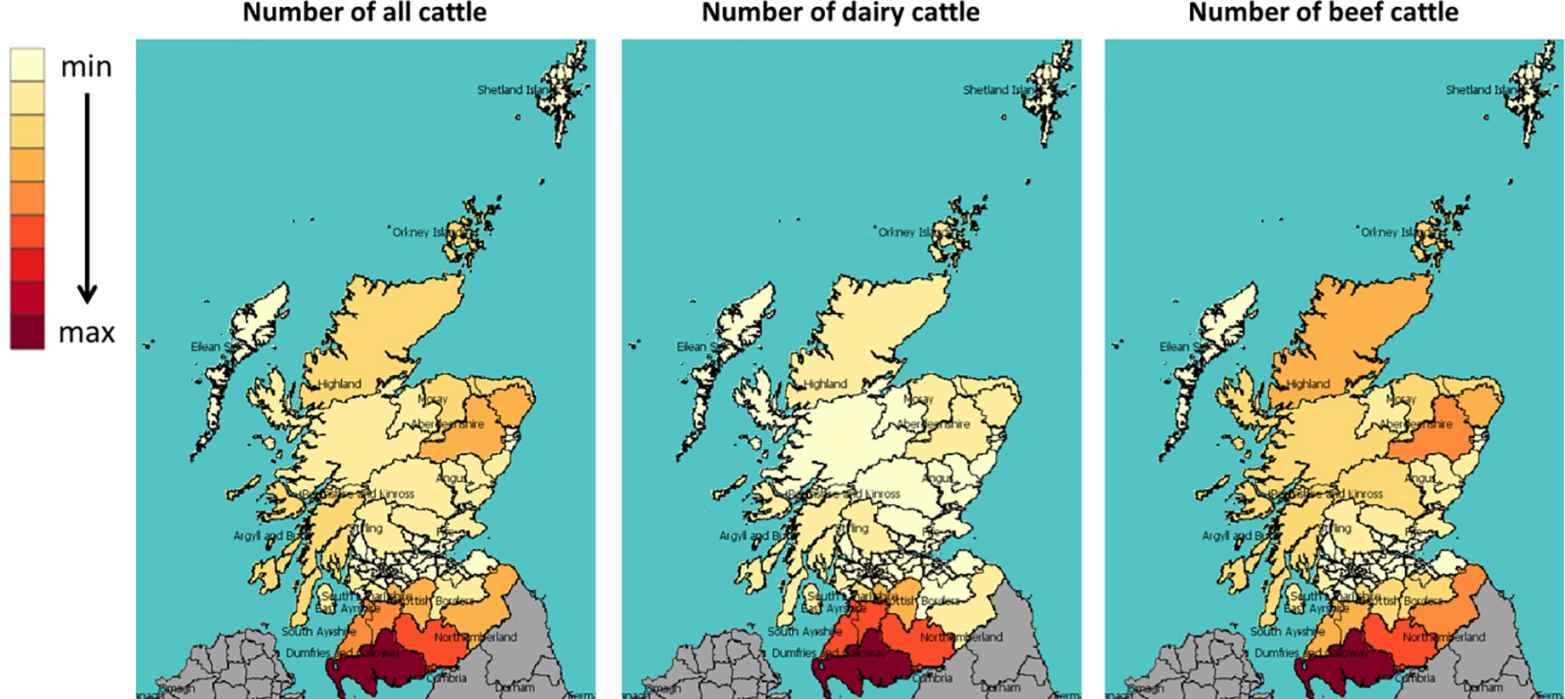
The number of all, beef and dairy cattle in each constituency in 2000.

**Fig 9:**
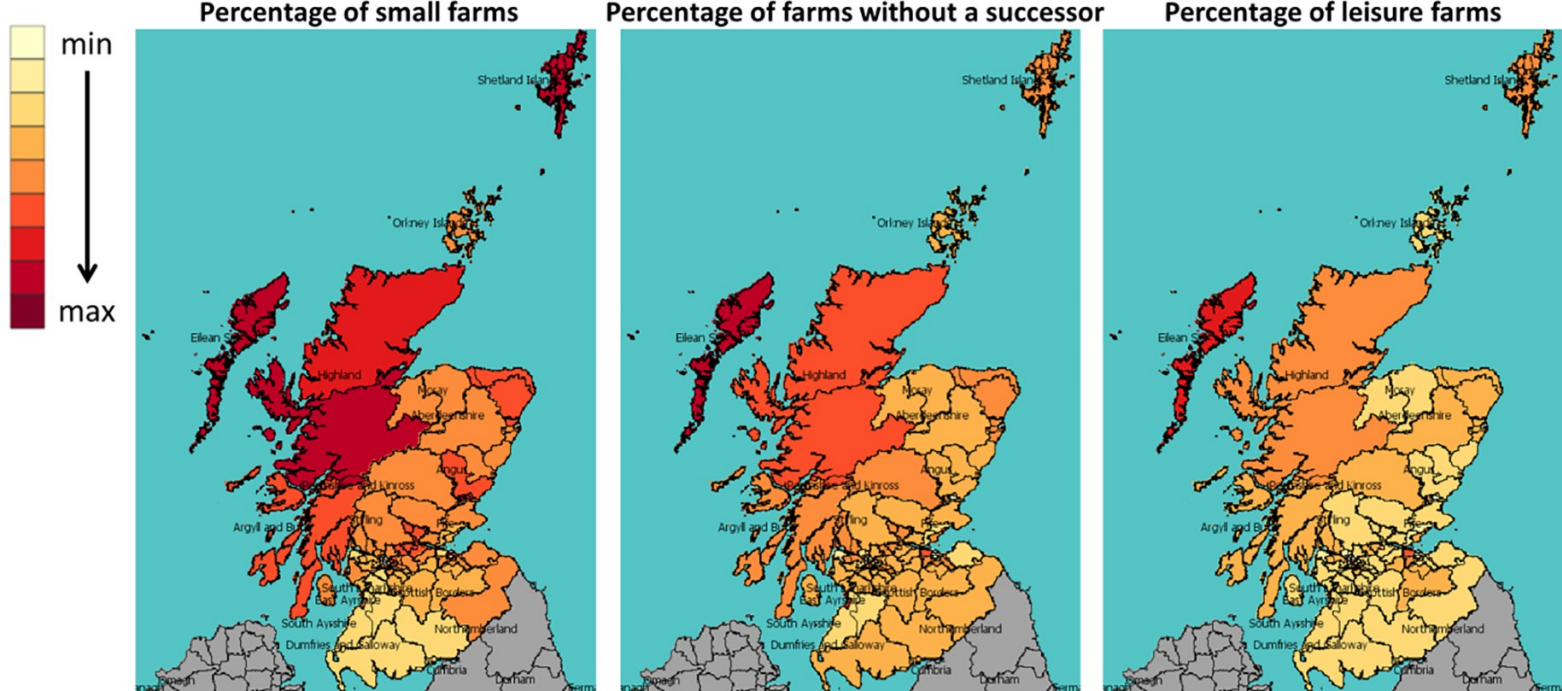
The percentage of small farms, farms without a successor and leisure farms in each constituency in 2000.

Fig 8 shows, from left to right, the numbers and geographical distribution of all, then dairy and beef cattle, and Fig 9 maps the percentages of small farms, farms without a successor and leisure farms across Scottish Parliament constituencies in 2000. The colour scheme shows the value in each constituency: The darker the shade, the higher the value. We see that, in general, constituencies in southwest Scotland have a larger number of cattle, especially dairy cattle; whereas constituencies in northwest Scotland (mainly the highlands and islands) have more small farms, farms without an immediate successor and leisure farms. For more information on the data behind Fig 8 and Fig 9, including the mean and standard deviation of the constituencies, please see the supporting information.

### 3.6. Input data

The input data include output and input price, average weight (beef) and milk yield (dairy) per cow, as well as the successor and off-farm income situation of each holding from 2000 to 2012. The data is input into the model in each time step (which represents a year in real life). The price and yield data are obtained from the Economic Report on Scottish Agriculture (ERSA), which is published annually by the Scottish Government [76]. The successor and off-farm income data are from the JAC and FSS from 2000 to 2012. Table 4 lists the input data of the model.

**Table 4:** Input data.

Variable	Entity	Data source (time period)	Input frequency
Beef price	Global	ERSA (2000–2012)	annual
Dairy price			
Average weight per beef cattle			
Annual yield per cow			
Hay price			
Straw price (both barley and oats)			
Successor?	Holding	JAC/FSS (2000–2012)	
Off-farm income?			

### 3.7. Submodel–Profit calculation

For the profit-seeking farms that produce the final beef and dairy product, in each period they calculate profit from selling the final product in the last period as a guideline to make the production decision in the following period. The profit of the farm depends on the price of the final product, the price of input such as feed and bedding, the weight of the beef cattle and the yield of the dairy cattle, and the area of barley, oat and grass the holding has. The calculation of profit is shown in the following equation,

$$\pi_{k}^{i}=p_{k}*yield_{k}*nCattle_{k}^{i}-p_{hay}*\max\left(0,\;p_{hay}*(feedPerHead_{k}-{feedPerHeadOwn}_{k}^{i})*nCattle_{k}^{i}\right)-p_{straw}*\max\left(0,\;p_{straw}*(strawPerHead_{k}-{strawPerHeadOwn}_{k}^{i})*nCattle_{k}^{i}\right)$$ (1)

where *k* is the type of cattle, which can be beef or dairy, and *i* is the holding. Therefore, $\pi_{k}^{i}$ is holding *i’s* profit from cattle type *k*, *p*_*k*_ is the price for beef or dairy, *yield*_*k*_ is the yield per head in terms of beef and milk production, and $nCattle_{k}^{i}$ is the number of beef or dairy cattle holding *i* owns. The global variables *feedPerHead*_*k*_ are the amounts of feed needed per beef and dairy cattle. We assume it is higher for dairy cattle due to the lower turnover and the longer time they are kept on farm. For the same reason, we assume that the value for the global variable *strawPerHead*_*k*_ is higher for dairy cattle.

Some holdings have grass and rough grazing areas where the cattle can graze. Hence the amount of hay (cattle feed) the farmers need to purchase depends on the area of grass and rough grazing a holding has. Since grass is better for grazing than rough grazing, we assume a different conversion rate to dry feed for grass and rough grazing areas. Similarly, some holdings grow barley and oats, the straw of which can be used for bedding. Therefore, the amount of straw the farmers need to purchase depends on the area of oats and barley that grows on their own land. Farms can also be self-sufficient in feed or straw, in which case the purchase for feed or straw is zero. The calculation of the feed and straw produced on farm are shown in the following equations,

$${feedPerHeadOwn}_{k}^{i}=(area_{grass}^{i}{*r}_{grass}+area_{rough}^{i}*r_{rough})*\frac{{feedPerHead}_{k}^{i}*nCattle_{k}^{i}}{{feedPerHead}_{k}^{i}*nCattle_{k}^{i}+{feedPerHead}_{-k}^{i}*nCattle_{-k}^{i}}$$ (2)

$${strawPerHeadOwn}_{k}^{i}=(area_{barley}^{i}{*r}_{barley}+area_{oats}^{i}*r_{oats})*\frac{{strawPerHead}_{k}^{i}*nCattle_{k}^{i}}{{strawPerHead}_{k}^{i}*nCattle_{k}^{i}+{strawPerHead}_{-k}^{i}*nCattle_{-k}^{i}}$$ (3)

where $area_{grass}^{i}$, $area_{rough}^{i},\;area_{barley}^{i}$, and $area_{oats}^{i}$ are holding *i’*s areas of grass, rough grazing, barley and oats respectively. The global variables *r*_*grass*_, *r*_*rough*_, *r*_*barley*, and *r*_*oats*_ are the conversion rate from grass area to hay, rough grazing area to hay, barley area to straw and oats area to straw respectively. The second terms on the right hand side of both equations $\frac{{feedPerHead}_{k}^{i}*nCattle_{k}^{i}}{{feedPerHead}_{k}^{i}*nCattle_{k}^{i}+{feedPerHead}_{-k}^{i}*nCattle_{-k}^{i}}$ and $\frac{{strawPerHead}_{k}^{i}*nCattle_{k}^{i}}{{strawPerHead}_{k}^{i}*nCattle_{k}^{i}+{strawPerHead}_{-k}^{i}*nCattle_{-k}^{i}}$ represent the proportion that the feed and straw produced on farm is given to beef and dairy cattle. Since dairy cattle require more feed and straw per head than beef cattle, the weight will be higher for dairy cattle.

## 4. The Brexit scenarios

We derive the Brexit scenarios from FAPRI (Food and Agricultural Policy Research Institute)-UK Brexit Report [77], which quantifies the impact of Brexit on commodity markets (including the beef and dairy market) using a partial equilibrium modelling framework. The report examines three alternative post-Brexit trade scenarios: 1) Bespoke Free Trade Agreement (FTA) with the EU, where the UK retains tariff and quota free access to the EU and vice versa; 2) World Trade Organisation (WTO) default Most Favoured Nation (MFN) tariffs, where the UK imposes the MFN tariffs to imports from the EU and vice versa; and 3) unilateral Trade Liberalisation (UTL), where the UK unilaterally waives all tariffs on imports from the EU and rest of the world. Similar scenarios and results are reported in Shrestha, Thomson [78]. Table 5 lists the estimated price changes in the Brexit scenarios.

**Table 5:** Brexit scenarios.

Scenario	no Brexit	FTA	WTO	UTL
Beef	0%	+3%	+17%	-45%
Milk and dairy	0%	+1%	+30%	-10%
Wheat (feed)	0%	-1%	-4%	-5%
Barley (feed)	0%	-1%	-5%	-7%

Davis, Feng [77] describe the three Brexit scenarios as follows: The first scenario, FTA, involves the least disturbances to the current trade arrangement. It assumes that the UK retains tariff and quota free access to the EU and vice versa. The UK also maintains EU tariff structure to the rest of the world. The only difference with the no Brexit scenario is a 5% trade facilitation costs on UK-EU trade. The second scenario, WTO, assumes that the MFN tariffs applied to UK exports for the EU and the EU imports into the UK. Moreover, there is an additional 8% trade facilitation costs on UK-EU trade. The WTO scenario describes the no-deal Brexit. The third scenario, UTL, represents a case where the UK unilaterally opens its borders and becomes free trade zone to the world. It assumes zero tariffs applied on imports to the UK from both the EU and the rest of the world. It also assumes MNF tariff applied to UK exports for the EU, as well as an 8% trade facilitation costs on UK-EU trade.

## 5. Results

In this section we show results from the model as used to select the combinations of drivers that reproduce the pattern on the disappearing middle. We then show the results for the four selected such combinations of drivers using the above Brexit scenarios, with overall trends first, followed by spatial distribution of effects.

### 5.1 Which processes? Using pattern-oriented modelling (POM) to select social processes

In section 2 we discussed several patterns and mechanisms that could have caused the phenomenon of disappearing middle, including the impact of 1) succession, 2) leisure farming (leisure), 3) diversification and 4) industrialization. The four mechanisms are Boolean parameters that one can turn on and off. When turned on, the mechanism will play a role in the farmer’s decision making, and when turned off, it will not. For example, when succession is turned on, a farmer will consider the issue succession when making a decision and adopt a different behaviour rule based on whether he or she has a successor; when succession is turned off, on the other hand, farmers will not include the issue of succession in their decision making process. In other words, succession will not affect farmer decision making.

**Fig 10:**
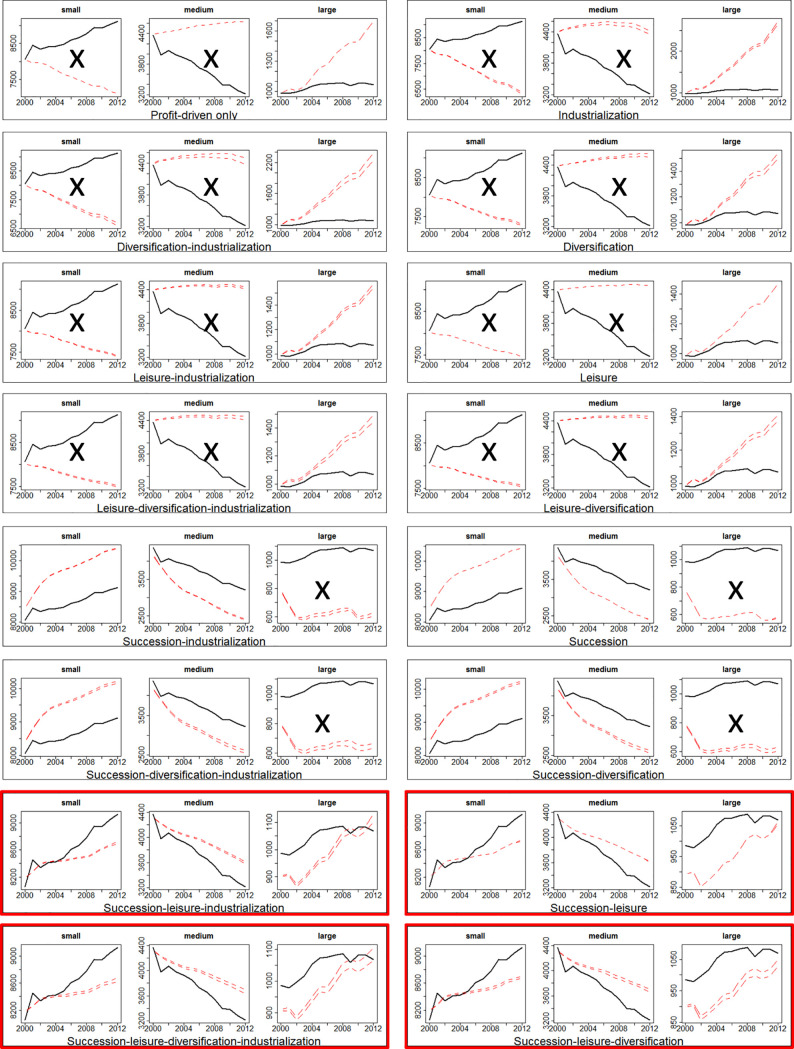
Model selection using pattern-oriented modelling (POM). Each of the sixteen frames shows the results for changes in numbers of small, medium-sized and large farms, after running the model with the labelled drivers switched on (“profit-driven only” means none are switched on). The graphs show the results from the model with red dashed lines (capturing minimum and maximum values from model runs) and the empirical data (from Fig 4) with a black solid line. The selected combinations are highlighted in red rectangles, and reproduce the observed pattern of increasing numbers of small and large farms as the number of medium-sized farms decreases. A black cross is placed where the simulated patterns does not match the empirical ones.

Although all four mechanisms (succession, leisure farming, diversification and industrialization) can be present in Scottish cattle farming, it is not clear which ones are necessary to cause the observed pattern of disappearing middle. To find out, we allowed each contested mechanism to be switched on and off, and ran the model on each of the sixteen combinations of the four mechanisms for 40 replications. We then matched the range of the outcomes against the data, which is shown in Fig 10. The analysis throughout this section was done in R, a free software environment for statistical computing and graphics [79].

We found out that, of the sixteen possible combinations of the four mechanisms, four have successfully reproduced the pattern of the disappearing middle (in red rectangle) in a qualitatively similar way. The four successful combinations are 1) succession-leisure-diversification-industrialization; 2) succession-leisure-diversification, but no industrialization; 3) succession-leisure-industrialization, but no diversification; and finally, 4) succession-leisure, but no industrialization or diversification. In other words, to reproduce the pattern of the disappearing middle, the issue of succession and leisure farming are necessary, but not industrialization or diversification, although they do not reverse the pattern. Models that do not include succession and leisure farming, on the other hand, have failed to reproduce the disappearing middle. They tend to overestimate the number of medium-sized and large farms and underestimate the number of small farms.

Grimm, Revilla [80] first introduced pattern-oriented modelling (POM) as a way to design, test and analyse agent-based complex systems. POM can be used to reduce uncertainties in model parameters by matching model results against multiple observed patterns. Researchers accept the parameters that can regenerate the multiple patterns simultaneously and reject those that do not. In our case, the disappearing middle phenomenon speaks to three related patterns: the increasing number of small farms, the decreasing number of medium sized farms, and the increasing number of large farms. The combined effects of the various mechanisms need to generate the three patterns simultaneously to be selected. In the following sections, we will look at the impact of Brexit in the four models that have passed POM. In other words, we will limit the analysis and discussion henceforth to the four models that have successfully replicated the disappearing middle phenomenon.

### 5.2 Not one Brexit: The influence of pre-Brexit trends

**Fig 11:**
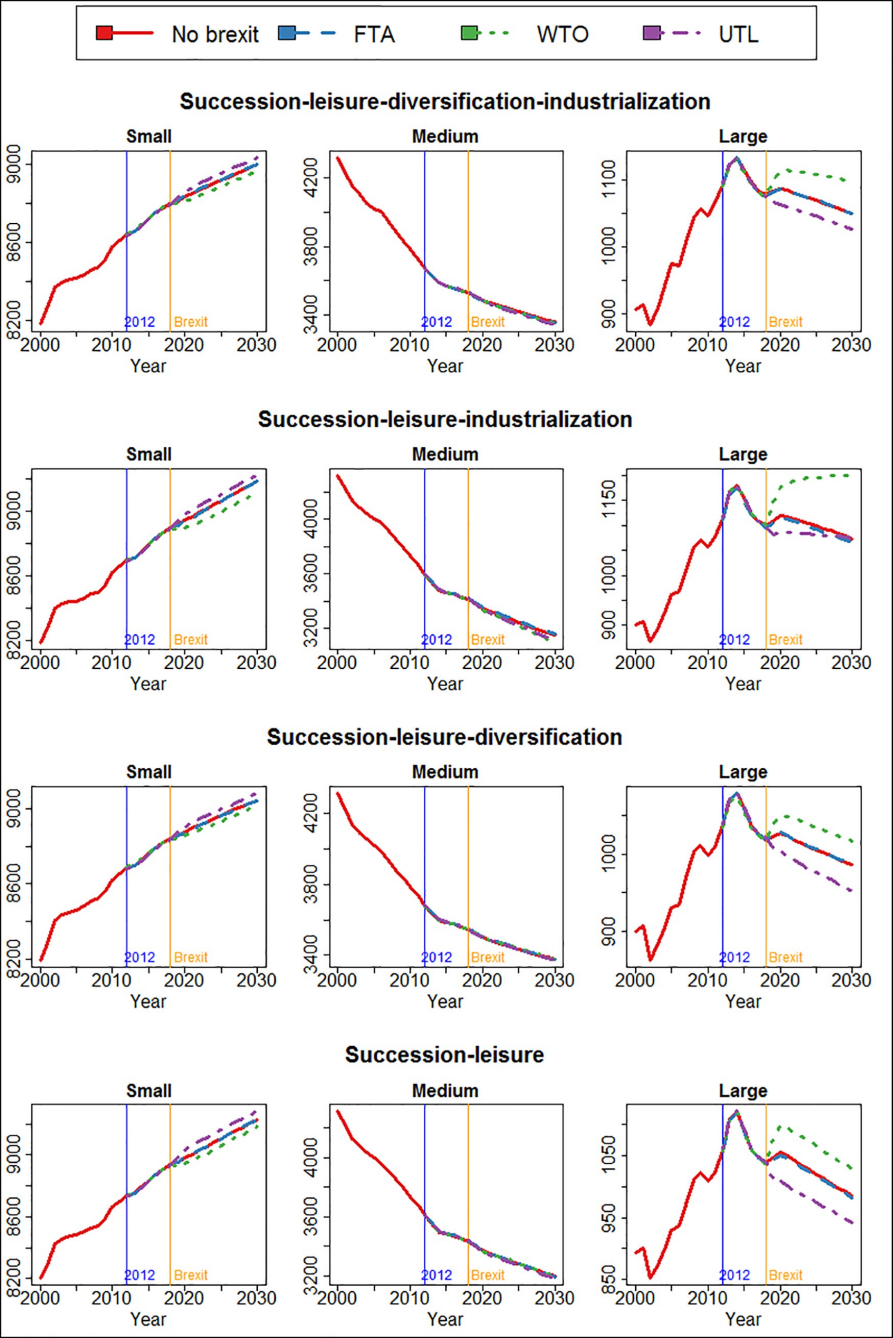
The number of small, medium and large cattle farms in the four Brexit scenarios predicted by the four models selected from POM.

Fig 11 shows the number of small, medium and large cattle farms in Scotland under the alternative Brexit scenarios and under different combinations of pre-existing local mechanisms (succession, leisure farming, diversification and industrialization). The model predicts that, before Brexit, the increasing trend for the number of large cattle farms is reversed. With no Brexit, the decreasing trend will simply continue. It is no surprise that Brexit scenarios have the largest impact on large cattle farms, because they are more responsive to price and profit than smaller ones, which are more likely to have income from off-farm employment or tourism, or are closing down due to lack of succession.

In the WTO scenario, because beef and dairy price will increase, the number of large cattle farms will increase significantly compared with the no Brexit and FTA (with marginal changes after Brexit) scenarios. The local preconditions determine to what extent the WTO arrangement will boost the number of large cattle farms. In the case of no industrialization, WTO Brexit will increase the number of large farms temporary, but does not alter the overall trend of decline of large farms. If, however, industrialization is present, the increasing in the number of large cattle farms will continue and the previous declining trend before Brexit will be reversed altogether.

In the UTL scenario, because the beef and milk price are much lower after Brexit, the number of large farms will be much lower than in other scenarios. Yet whether industrialization exists in the local area before Brexit still determines the long-term trend of large cattle farms. If industrialization exists, the number of large cattle farms will stabilize after the initial shock of a UTL Brexit; otherwise the decline will continue. Studies have found that the industrialization or intensification of agricultural production can have a profound impact on the structure and the nature of farms by changing farms’ scale, efficiency, vulnerability, requirement of inputs and land, and the way they behave [60, 81, 82]. Thus, it is not entirely surprising that we find here industrialization could potentially reverse the trend and impact from Brexit.

Interestingly, Brexit does not seem to affect the decline of medium-sized cattle farms. Brexit or not, the existing trend of “the disappearing middle” seems to continue regardless. The influence of Brexit on small farms is small, too, and does not change the general increasing trend. However, the impact is distinguishable by the Brexit scenarios: the UTL arrangement increases the number of small cattle farms, whereas the WTO arrangement decreases it. The reason Brexit does not affect small or medium-sized farms as much is because other mechanisms such as succession, leisure farming and diversification dominate development of small farms, compared with which Brexit plays only a marginal role.

**Fig 12:**
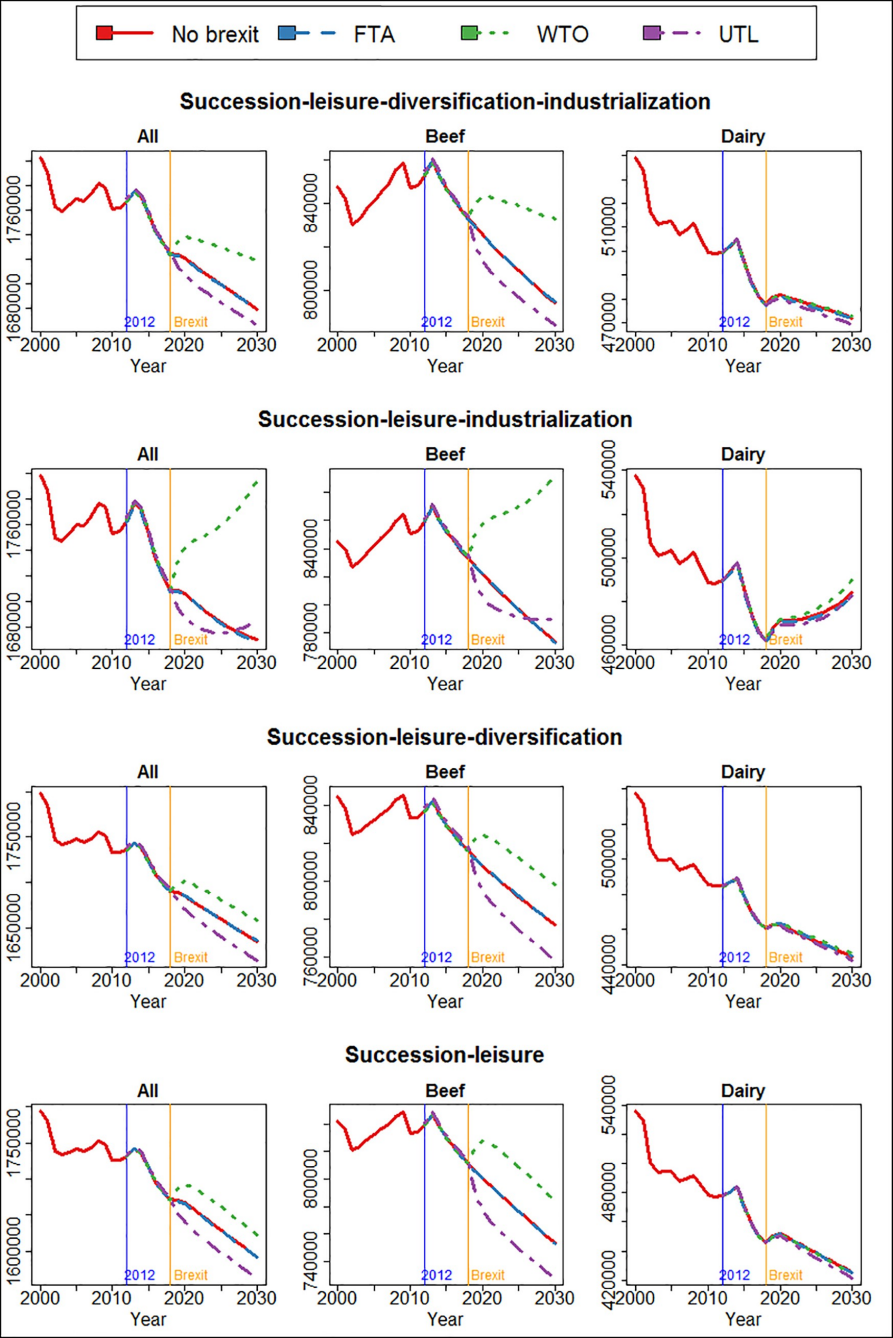
The number of all, beef and dairy cattle in the four Brexit scenarios predicted by the four models selected from POM.

Fig 12 shows the number of all cattle, as well as beef and dairy cattle in Scotland. All cattle include beef, dairy and mixed types. Since the WTO arrangement means higher output price (beef and dairy), lower input price (wheat and barley) and thus more profit, the number of cattle is significantly higher than without Brexit or in the FTA scenario. Conversely, in the UTL scenario, because the price for beef and dairy will be significantly lower, the number of all cattle as well as beef and dairy cattle will be lower than without Brexit or FTA.

More importantly, the impact of Brexit on the number of cattle is highly sensitive to the other mechanisms. As we previously discussed, the number of cattle in Scotland has been in a long-term trend of decline since the 1970s. Without industrialization, we find that even the most favourable (to the cattle sector) WTO scenario is insufficient to reverse the declining trend. Only in the second case where succession, leisure farming and industrialization are present but not diversification, is the general trend of decline reversed. In that case, a WTO arrangement will increase the number of cattle so much that it exceeds the initial level and starts to grow. Interestingly, even in the unfavourable scenario of UTL, the declining trend seems to be reversed and the number of cattle stabilized towards the end of the simulation. In all other three cases, on the other hand, although Brexit scenarios have produced temporary shocks to the number of cattle, they do not alter the general trend in the long term. The interactions between Brexit and the other mechanism are important: The model could predict completely opposite Brexit impacts (case 2 vs. the rest) based on which mechanisms are present simultaneously.

### 5.3 Not one Brexit: The influence of location

This section shows the impact of Brexit on Scotland’s 73 constituencies. Throughout the rest of this section, we will use the following visualization: A shade of green means a percentage increase in the number of cattle between 2019 (Brexit) and 2030; whereas a shade of purple means a decrease. The darker the shade, the higher the percentage increase or decrease.

**Fig 13:**
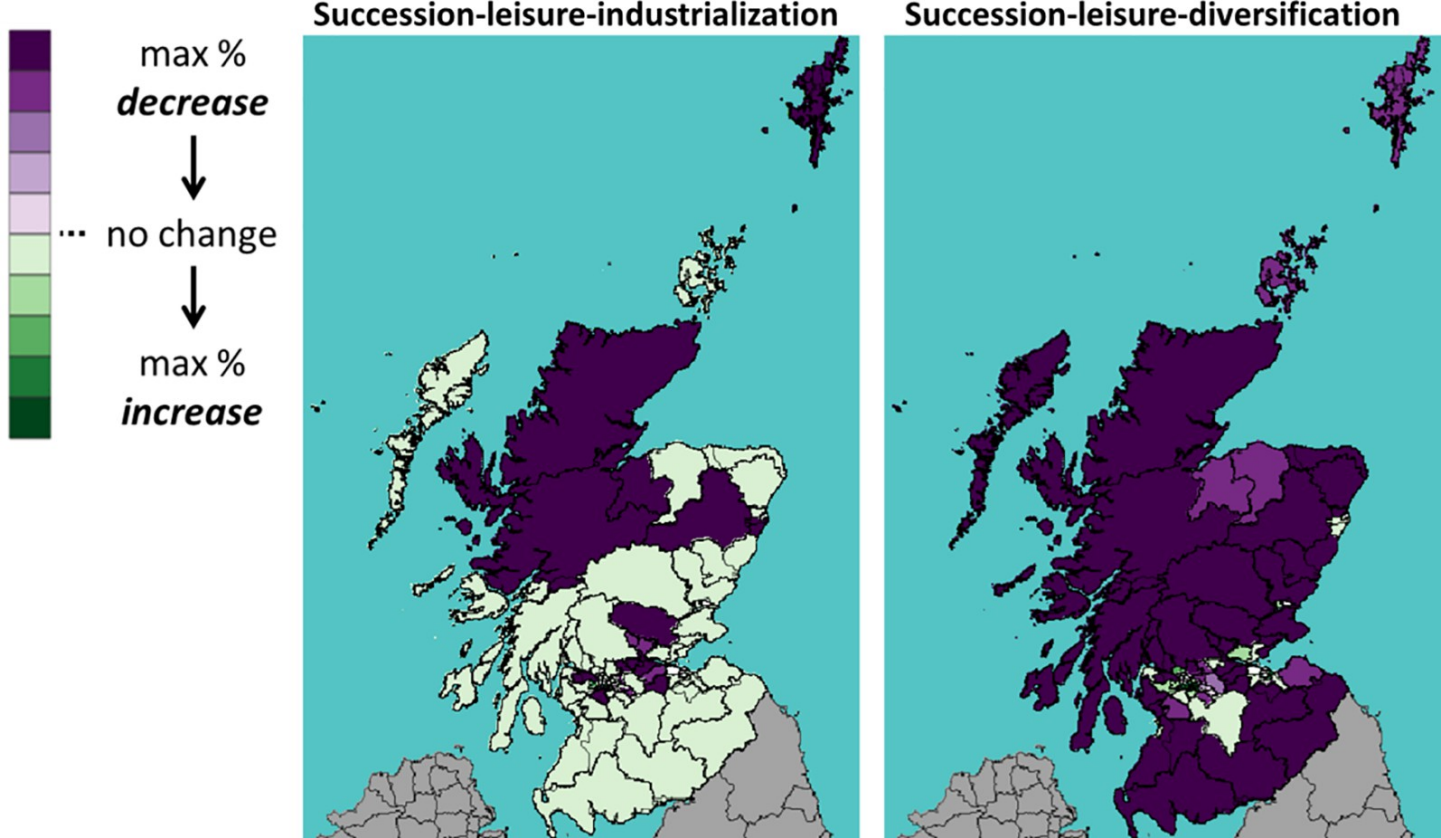
Percentage change in the number of *all* cattle under WTO scenario.

**Fig 14:**
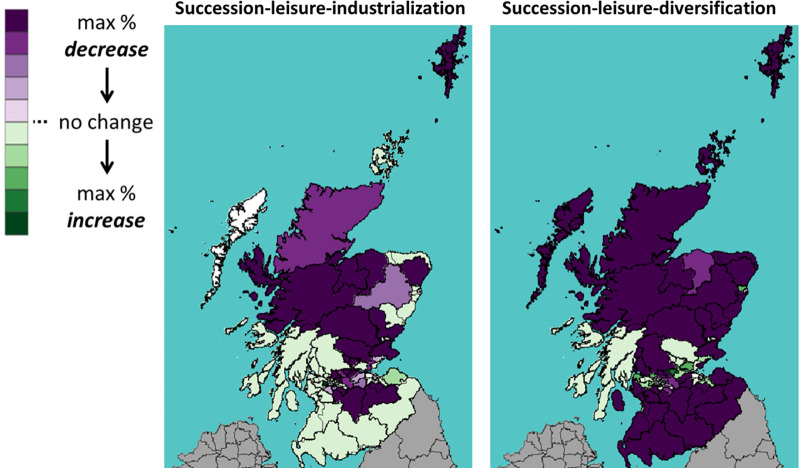
Percentage change in the number of *dairy* cattle under WTO scenario.

**Fig 15:**
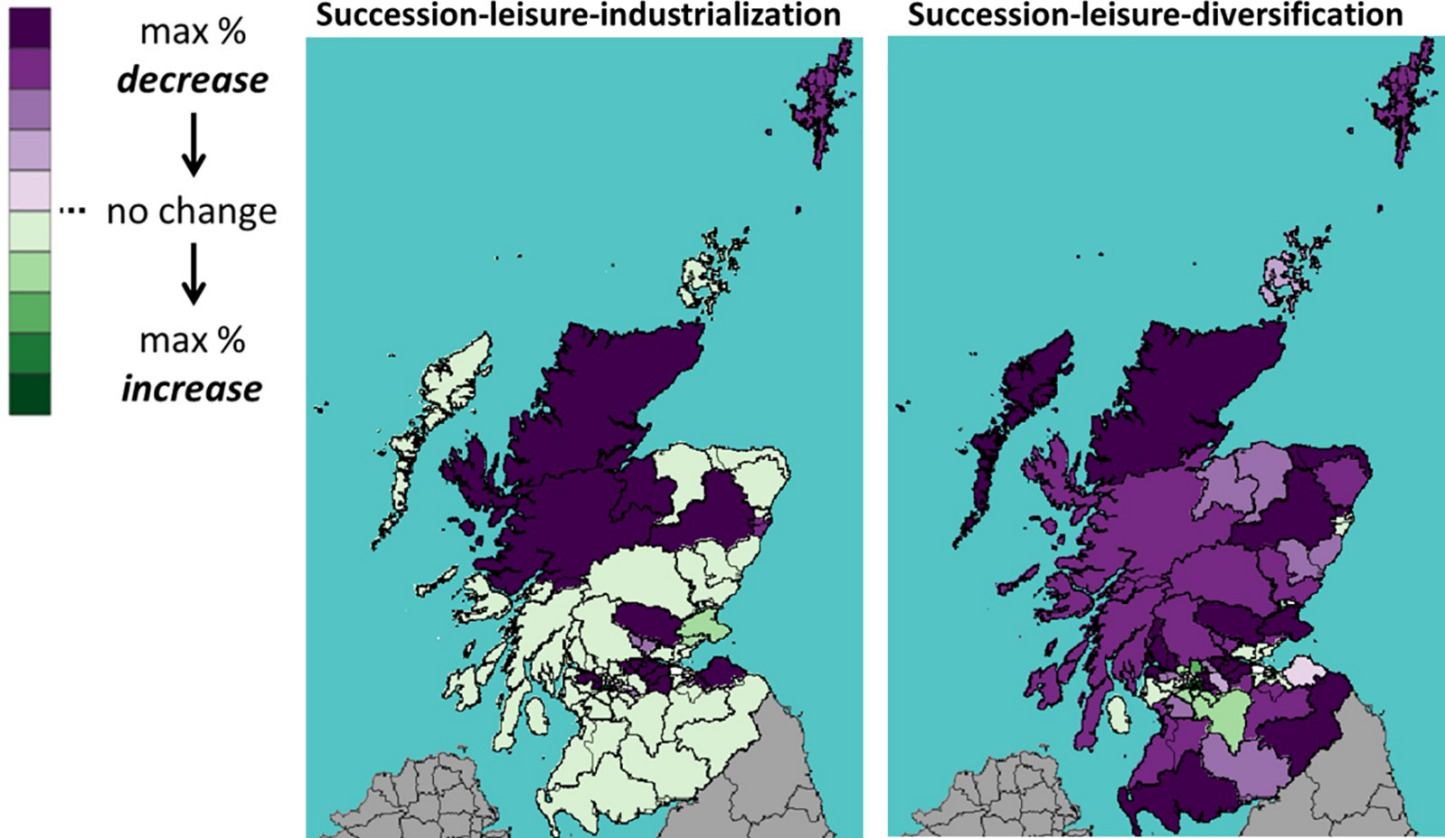
Percentage change in the number of *beef* cattle under WTO scenario.

Figs 13–15 show the percentage change of all, dairy and beef cattle under a WTO Brexit, which is considered favourable to Scottish cattle farming. We see that the Brexit impact is very different on the areas in the north (highland and islands), the middle (the central belt) and the south. In the case with industrialization (left panel), the number of cattle increase in most southern constituencies and decrease in most northern ones. In the case of diversification (right panel), the central belt and Aberdeen (in northeast) see their number of cattle increases, whereas the rest sees a decrease.

**Fig 16:**
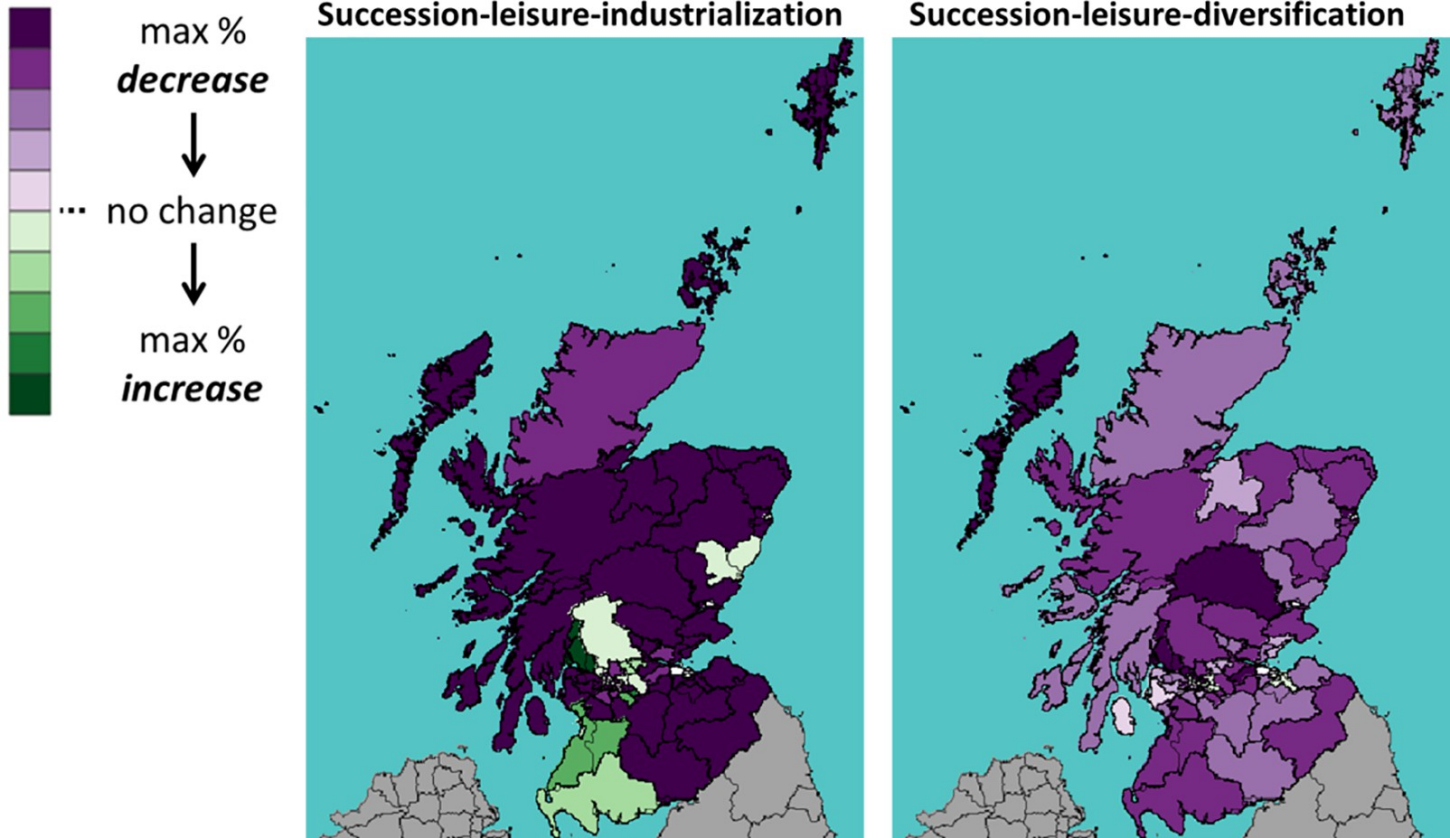
Percentage change in the number of *all* cattle under UTL scenario.

**Fig 17:**
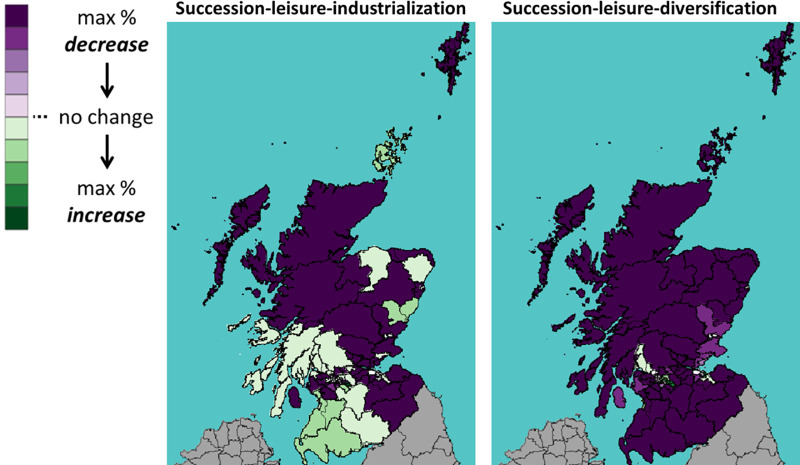
Percentage change in the number of *dairy* cattle under UTL scenario.

**Fig 18:**
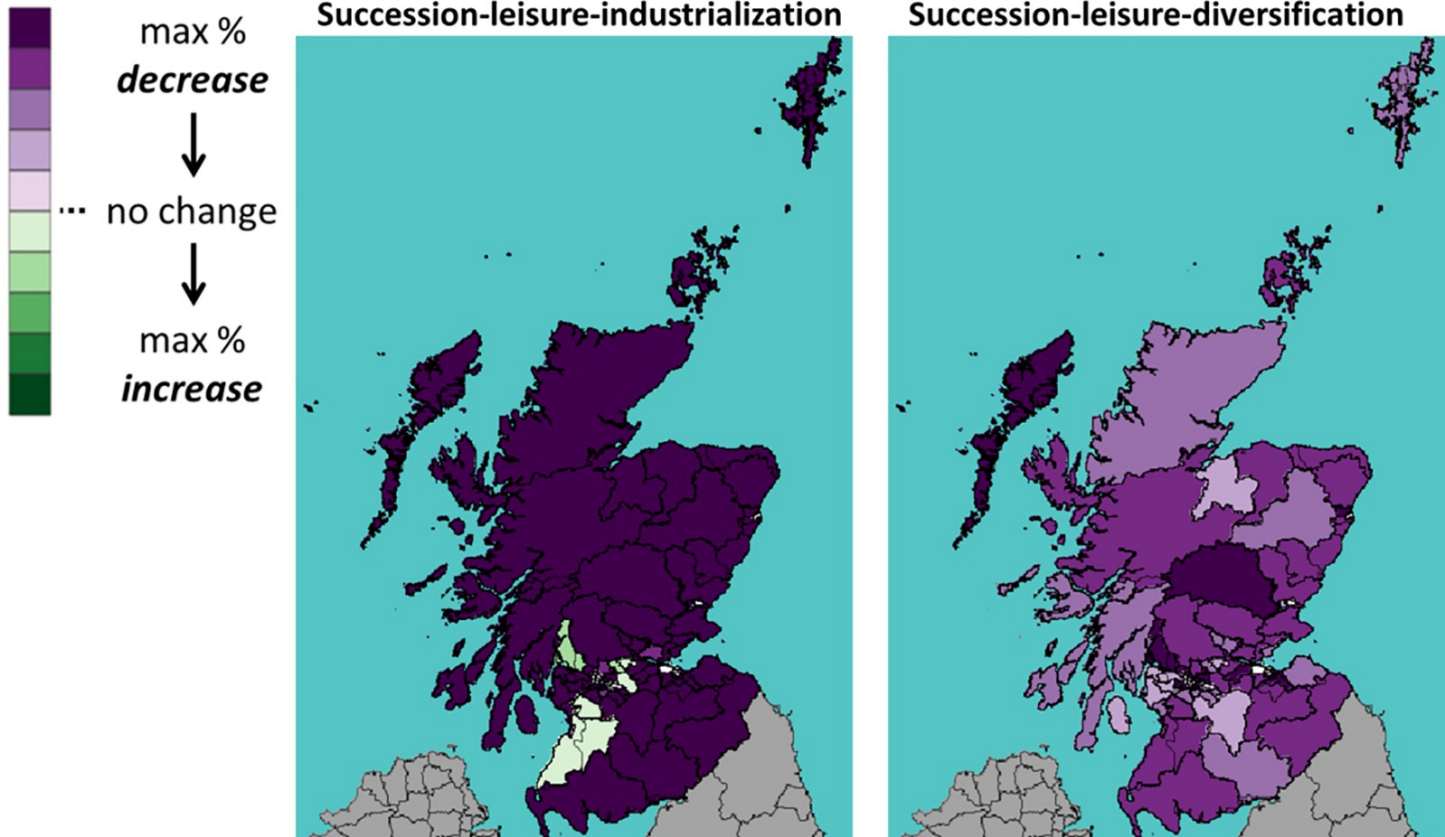
Percentage change in the number of *beef* cattle under UTL scenario.

Figs 16–18 show the percentage change in the number of all, dairy and beef cattle after a UTL Brexit, a scenario considered unfavourable to Scottish cattle farming. With industrialization (left panel), the number of cattle in southwest constituencies still increases even after an unfavourable Brexit. Without industrialization (right panel), however, the number of cattle decrease in most constituencies.

## 6. Discussion

The results have shown that local contexts, trends and mechanisms that exist before Brexit are important and can potentially change the direction of Brexit’s impact. For the 73 constituencies and the 13,406 cattle farms in Scotland, each will have a different Brexit story. A large, industrialized dairy farmer in southwest Scotland, for example, will probably still grow even in the least favourable Brexit scenario, whereas a small beef farmer in northern Scotland will struggle even in the most favourable Brexit scenario. For others with off-farm income or income diversification activities, Brexit may not even be a major factor. Therefore, depending on the type and size of the majority of cattle farms in each constituency, political representatives there will also face very different issues, needs and requests from cattle farmers after Brexit. Again, for each constituency and the politicians representing it, Brexit does not have the same impact.

The results show the observed trend of polarization among Scottish cattle farms continuing until Brexit, and we were curious to find out if Brexit could potentially alter it. However, we found that the decline of medium-sized farms is persistent and more or less unaltered by Brexit. The reason is that the two main forces behind the trend: the lack of succession and the rise of leisure farming are more fundamental than can be moved by the market changes brought about by Brexit. Brexit may influence succession and leisure farming in some non-market ways, but it is unclear how.

Policy frameworks devoid of local context and processes can easily lose meaning and insight. Ostrom [83] talks about such oversimplified policy frameworks in the context of common-pool resource management, in which she argued that the local context is extremely important in determining the impact and effectiveness of any policy, and that the same concept such as “common-pool resources” and “human interactions” can have very different meanings and implications in different contexts. Having just one behavioural mode, one agent type and two contrasting systems and scenarios is therefore insufficient. Policy frameworks need to be more flexible in order to represent the complex reality at multiple levels. This study provides an example of a policy framework that takes a complex system approach that incorporates a higher level of interactions and heterogeneity.

This study includes four combinations of mechanisms or processes that could possibly lead to the disappearing middle phenomenon. Given the data we used in this study, we could not rule them out using the POM approach [80]. We will need more data to find out what exactly is contained in the current trends. Such information is more likely to come from a survey on farmers’ attitudes and intentions (such as in Sutherland, Toma [84]), than standard census data that focus more on physical attributes and existing activities. We should also keep in mind that the situation is likely to change over time. New types of farming still unknown to us may arise while old ones die out. Current trends may be reversed or take on new forms. The “not one Brexit” notion has a temporal aspect as well: Brexit until 2030 may be very different than Brexit in the longer run, which, given all the uncertainties, is even harder to predict.

The study has made several simplifications. The impact of Brexit is largely limited to market price (although this already considers additional administrative costs at the borders); whereas in real world, it may have a wider influence socially and culturally, such as the Brexit impact on agricultural tourism, immigration and income level. The farmers’ decision-making procedure (Fig 7) is highly sequential and linear; whereas in real world, a decision made down the line may lead to reconsideration of a previous decision. There are policy interventions such as changes to agricultural policy that we have not included in the study. The selling and buying of land have been reduced to the change in the owner (derived from the census data). Other potential complications that we did not consider include the transport network, the social networks among farmers and changes in wages and labour costs.

In this study, Industrialization is represented by the farm being run by a professional manager rather than the farm owner and the family, which is supported by literature [60] and (indirect) empirical evidence (Fig 3). However, we understand that there are many alternative ways to characterize and define an industrialized farm. One is by the adoption of certain technology (such as a milking robot). We did not choose this definition because the cattle farms included in the study are so diverse (e.g. beef vs. dairy; breeder vs. raiser vs. finisher) that the technology they use to expand and industrialize the farms will also be very different. There is not a clear ‘cut-off’ technology that can separate the industrialized farms from the family-run ones. We have not considered the access to capital and credit in the process of industrialization, because most farms in Scotland have access to capital, loans and other financial assistance should they need to purchase machinery and equipment, so it is not a major concern in farmers’ decision making [85]. As we have discussed before in Section 2.4, the definition of industrialized farms in existing literature tend to be descriptive and qualitative, such as using a manufacturing approach or the high level of specialization. There is not an easy and clear-cut way to distinguish industrialized and non-industrialized farms, either by size, managerial structure or the technology used. Also, we believe that the definition will differ by country, region and the type of farming.

Because we focus on the farmer’s decision to change existing herd size (not to decide on its level), we have not included certain geographical factors in the decision making algorithm, such as distance to market, soil quality and climate because they do not change (or change very little) over the course of the study, so will not influence the farmer’s decision to change the herd size. However, there might be interactions between these factors and Brexit. For example, Brexit may change the impact and importance of certain geographical factors; it may alter the development of agricultural tourism; and it may change the level of income. We did not consider these potential interacting effects because so far it is unclear if and how these effects will take place, and little evidence exists to support assumptions either way.

The definition of a ‘cattle farm’ in this study (farms that has at least one head of cattle in the year 2000) means that we include more than 1500 (out of 13406 cattle farms) very small farms with very few (e.g. less than 10) cattle. Although some of those small farms with a few cattle may not be regarded as ‘proper’ cattle farms in a conventional definition, we believe it is important that we include them in this study, because they may reflect the emerging trends that drive the current phenomenon. The farms with few cattle may be the newly arrived leisure farmers from the city; or the retired farmers who have been gradually shrinking their herd due to lack of succession; or the farms diversified into tourism that keep a few cattle for their touristic values. Although small, these farms may reflect those ongoing processes, and thus it is important that the model and the analysis in the paper include them.

From a modelling perspective, one of the most interesting aspects of our results is the fact that the same patterns of macro-level behaviour can be achieved with different ontological assumptions about the drivers thereof. Further, ontological assumptions generating matching patterns do not have consistent implications for the impact of Brexit. The work addresses emerging concerns in modelling pertaining to structural validation and uncertainty [86–88]. To our best knowledge, this paper is one of the first to demonstrate the issue directly and explicitly. Under a classical modelling paradigm, Ockham’s Razor means that the simplest model generating observed behaviour is preferred. In this case, that would be the succession-leisure combination, and modelling heuristics would then ignore any potential effects of diversification or industrialization. Our results show how such simplification could have effects on conclusions with regional implications. “More than one Brexit” thus applies to modelling the impacts of Brexit as much as it does to the heterogeneity in context of the farmers affected by it.

## 7. Concluding remarks

We develop an empirical agent-based model to investigate the impact of Brexit on Scottish cattle farms, while acknowledging that long before Brexit, changes have been ongoing among Scottish cattle farms that have led to the polarization of cattle farms and the decline of medium-sized farms. From empirical evidence, existing literature, and local expert knowledge, we identify four factors and drivers that could cause the disappearing middle phenomenon: the lack of succession, the rise of leisure farming, diversification and industrialization. We ask the following research questions: How does a major national event like Brexit interact and interfere with local context, trends and processes? To what extent does the impact of Brexit depend on the trends and conditions that exist before Brexit? And which types of farms will be mostly impact upon by Brexit?

We find that pre-Brexit conditions could lead to very different conclusions regarding the impact of Brexit. In particular, whether industrialization is a dominant trend locally can significantly alter the size and direction of Brexit impact. We show the Brexit effect is more pronounced on large cattle farms, which are predominantly profit-seeking, than small and medium ones, many of which are diversified, living on off-farm income or closing down due to lack of succession. As for the phenomenon of the disappearing middle, we find that Brexit will not alter or reverse the long-term trend, mainly because the mechanisms behind it such as lack of succession are more fundamental and persistent than can be moderated by the market changes brought about by Brexit. Finally, the impact of Brexit varies across regions: some constituencies will be more resilient than others; the winners and losers will also depend on the Brexit scenario and the type of cattle.

When talking about the impact of Brexit, we need to ask, “Brexit for whom?”, as Brexit would mean very different things to different people and groups. There is no one Brexit for all. The analysis of Brexit, therefore, should take into account the heterogeneity and the complex local context and social environment under which Brexit occurs. Although it might make policy analysis more challenging and the results less clear-cut, we should not shy away from the complexity that is an inevitable part of life. Instead of fitting the world into simple system frameworks and refuse to see the evidence when it does not fit, as Ostrom [83] correctly put it, we need to develop policy frameworks that can deal with complexity and so allow us to analyse policy in a more relevant and meaningful way.

## Supporting information

S1 Appendix (DOCX)
