# Correction: Not one Brexit: How local context and social processes influence policy analysis

**DOI:** 10.1371/journal.pone.0317532

**Published:** 2025-01-09

**Authors:** The PLOS ONE Editors

## Notice of Republication

This article was republished on October 17, 2024, to address an issue identified post-publication. Please download this article again to view the correct version.

The article’s Data Availability statement is updated to: The dataset used for this study is the Scottish Government (2010–2012) June Agricultural Census which was collected and owned by the Scottish Government and cannot be shared under a CC BY 4.0 license. Requests for access to the dataset should be submitted to agric.stats@gov.scot.

Summary reports of these data are available in [2–4].
