# Supplementary material for: Not one Brexit: How local context and social processes influence policy analysis
Source: PLoS One. 2018 Dec 17;13(12):e0208451. doi: 10.1371/journal.pone.0208451 (PMC6296738; doi:10.1371/journal.pone.0208451)
Supplement: S1 Appendix — (DOCX) [file pone.0208451.s001.docx]

# Appendix

## Cluster analysis of cattle farms

We did a cluster analysis on cattle farms by the number of cattle in different cattle categories between 2000 and 2012. The data is from June agricultural census. The cattle categories on which the cluster analysis is based are listed in Table 1.

S1 Table Variables used in cluster analysis

| **Item index in census** | **Item description** |
| --- | --- |
| ITEM100 | Dairy cows and heifers in milk |
| ITEM101 | Beef cows and heifers in milk |
| ITEM102 | Dairy cows in calf but not in milk |
| ITEM103 | Beef cows in calf but not in milk |
| ITEM104 | Dairy heifers 2 years old and over in calf for the first time |
| ITEM105 | Beef heifers 2 years old and over in calf for the first time |
| ITEM106 | Dairy heifers under 2 years old in calf for the first time |
| ITEM107 | Beef heifers under 2 years old in calf for the first time |
| ITEM108 | Bulls for service aged two years old and over |
| ITEM109 | Bulls for service aged between 1 and 2 years old |
| ITEM110 | Male cattle aged 2 years and over |
| ITEM111 | Female dairy cattle aged 2 years and over for breeding |
| ITEM112 | Female beef cattle aged 2 years and over for breeding |
| ITEM113 | Female cattle aged 2 years and over not for breeding |
| ITEM114 | Male cattle aged between 1 and 2 years |
| ITEM115 | Female dairy cattle for breeding aged between 1 and 2 years |
| ITEM116 | Female beef cattle for breeding aged between 1 and 2 years |
| ITEM117 | Female cattle not for breeding aged between 1 and 2 years |
| ITEM118 | Male cattle aged between 6 months and 1 year |
| ITEM119 | Female cattle aged between 6 months and 1 year |
| ITEM120 | Male cattle aged under 6 months old |
| ITEM121 | Female cattle aged under 6 months old |
| ITEM122 | Total cattle |

We used the *k*-means method and plotted the within group sum of squares against the number of clusters (Fig 1). We chose three clusters because it is where the curve starts to flatten, and because it corresponds to the three size groups (small, medium and large) that we use in the study.


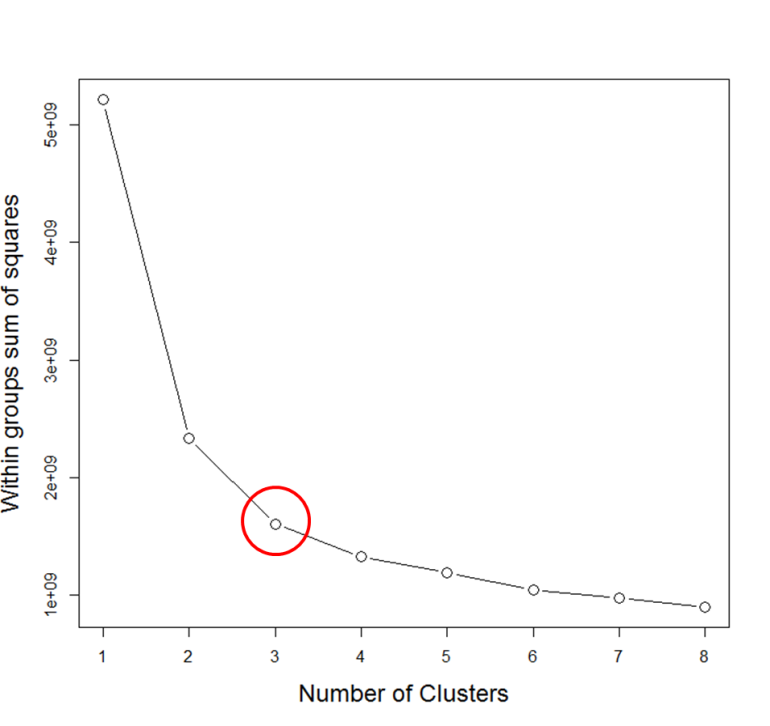


S1 Fig Clustering result and the selection of the number of clusters

The mean values of the three groups from the cluster analysis are listed in Table 2. The results shows that cattle farms are clearly clustered around three size groups: group 1 are large farms, group 2 are medium farms and group 3 are small farms. In almost all categories, large farms on average have at least twice as many cattle as do medium farms; whereas small farms only has a fraction of what large farms have on average. In terms of the number of farms in each group, the opposite is true: two thirds of the farms are small, while less than 10% of the farms are large. We use the average of the smallest large farm and the largest medium farm (368) as the cut-off between large and medium farms; and the average of the smallest medium farm and the largest small farm (131) as the cut-off between medium and small farms.

S2 Table Mean values of groups from cluster analysis

| **Item description** | **avg. group 1** | **avg. group 2** | **avg. group 3** |
| --- | --- | --- | --- |
| Dairy cows and heifers in milk | 59.6 | 22.2 | 1.1 |
| Beef cows and heifers in milk | 92.0 | 46.1 | 8.7 |
| Dairy cows in calf but not in milk | 8.5 | 3.4 | 0.2 |
| Beef cows in calf but not in milk | 20.5 | 11.2 | 2.7 |
| Dairy heifers 2 years old and over in calf for the first time | 9.2 | 3.6 | 0.2 |
| Beef heifers 2 years old and over in calf for the first time | 7.3 | 3.7 | 0.9 |
| Dairy heifers under 2 years old in calf for the first time | 6.7 | 1.9 | 0.1 |
| Beef heifers under 2 years old in calf for the first time | 3.2 | 1.6 | 0.3 |
| Bulls for service aged two years old and over | 4.1 | 2.1 | 0.4 |
| Bulls for service aged between 1 and 2 years old | 1.0 | 0.5 | 0.1 |
| Male cattle aged 2 years and over | 11.0 | 4.3 | 1.3 |
| Female dairy cattle aged 2 years and over for breeding | 1.7 | 0.9 | 0.1 |
| Female beef cattle aged 2 years and over for breeding | 4.5 | 2.3 | 0.8 |
| Female cattle aged 2 years and over not for breeding | 6.6 | 2.5 | 0.6 |
| Male cattle aged between 1 and 2 years | 53.2 | 23.0 | 5.7 |
| Female dairy cattle for breeding aged between 1 and 2 years | 14.0 | 5.0 | 0.3 |
| Female beef cattle for breeding aged between 1 and 2 years | 14.1 | 6.3 | 1.6 |
| Female cattle not for breeding aged between 1 and 2 years | 39.5 | 15.5 | 3.2 |
| Male cattle aged between 6 months and 1 year | 27.1 | 12.1 | 1.8 |
| Female cattle aged between 6 months and 1 year | 30.2 | 13.3 | 1.8 |
| Male cattle aged under 6 months old | 45.2 | 20.7 | 3.7 |
| Female cattle aged under 6 months old | 47.7 | 21.4 | 3.7 |
| **Total cattle** | **506.7** | **221.1** | **36.9** |
| **Number of holdings between 2000 and 2013 (%)** | **13643 (7.8%)** | **48372 (27.8%)** | **112263 (64.4%)** |

## June Agricultural Census (JAC) and EU Farm Structure Survey (FSS)

Most of the data collected in JAC are required by the Statistical Office of the European Communities, specifically Council Regulation 1165/2008. This information is collated by DEFRA for submission at member state (UK) level. It is conducted annually by the Scottish Government’s Rural and Environmental Science Analytical Services division (RESAS). Data are requested from all holdings who submitted a Single Application Form (SAF) in the previous year, together with some other large businesses that would not be eligible for support payments. A sample of holdings which didn’t submit a SAF or who didn’t return a form in the previous year are also sent a census form. There are a total of 51,356 registered agricultural holdings providing a virtually complete coverage of agricultural activity in Scotland. A full census is not conducted as this would place an unnecessary burden on farmers. For the selected holdings that are surveyed, not all farmers return data. Gaps in the holding-level data are ‘maintained’ by producing estimates by a process referred to as ‘imputation’.

The FSS collects information on the structural characteristics of the agricultural holdings (land use, livestock and labour force) and is carried out by all European Member States every 10 years as an agricultural census, with two or three additional, intermediate sample surveys carried out in-between. In Scotland the FSS is conducted at the same time as the June Census by way of additional questions in that year’s Census form. Common grazing is not included in either the JAC or FSS.

## Distribution of cattle farms across Scottish Constituencies in 2000

S3 Table Number of all, beef and dairy cattle in Scottish constituencies in 2000

|  | Number of all cattle | | Number of beef cattle | | Number of dairy cattle | |
| --- | --- | --- | --- | --- | --- | --- |
| Percentile | mean | sd \ mean | mean | sd \ mean | mean | sd \ mean |
| 0-20% | 781 | 0.85 | 272 | 1.03 | 292 | 1.04 |
| 21-40% | 4514 | 0.25 | 2000 | 0.41 | 1627 | 0.26 |
| 41-60% | 11038 | 0.2 | 4602 | 0.25 | 3715 | 0.26 |
| 61-80% | 35383 | 0.32 | 17768 | 0.46 | 9407 | 0.24 |
| 81-100% | 104556 | 0.49 | 48777 | 0.41 | 33462 | 0.74 |

S4 Table Percentage of small, medium and large farms in Scottish constituencies in 2000

|  | % of small farms | | % of medium farms | | % of large farms | |
| --- | --- | --- | --- | --- | --- | --- |
| Percentile | mean | sd \ mean | mean | sd \ mean | mean | sd \ mean |
| 0-20% | 0.26 | 0.68 | 0.11 | 0.93 | 0 | 3.74 |
| 21-40% | 0.47 | 0.07 | 0.32 | 0.08 | 0.04 | 0.36 |
| 41-60% | 0.56 | 0.03 | 0.37 | 0.05 | 0.07 | 0.09 |
| 61-80% | 0.62 | 0.04 | 0.45 | 0.06 | 0.1 | 0.1 |
| 81-100% | 0.84 | 0.17 | 0.64 | 0.28 | 0.26 | 0.93 |
